# Sol–gel synthesis and comprehensive characterization of MgO nanostructures: structural, optical, and dielectric insights

**DOI:** 10.1038/s41598-026-44397-5

**Published:** 2026-04-13

**Authors:** Naglaa AbdelAll, Asmae Mimouni, Abdalrahman M. Rayan, Ghada A. Khouqeer, Mohamed Asran Hassan, Mahrous R. Ahmed

**Affiliations:** 1 Physics Department, Faculty of Science, Assiut University, Assiut, 71516 Egypt; 2 Physics Department, Faculty of Science, Imam Mohammad Ibn Saud Islamic University (IMSIU), 11564 Riyadh, Saudi Arabia; 3 Physics Department, Faculty of Science, Sohag University, Sohag, 82524 Egypt

**Keywords:** MgO nanoparticles, Sol–gel, Energy gap, Cole–Cole, FTIR, SEM, Materials science, Physics

## Abstract

MgO nanostructures synthesized via the sol–gel method were examined for their structural, microstructural, optical, and dielectric properties. XRD indicated a crystalline cubic phase with an average crystallite size of approximately 30–50 nm, corroborated by TEM findings of quasi-spherical to polyhedral morphologies. Mapping of electron density distribution validated the face-centered cubic structure, while the modified Scherrer, Williamson-Hall, size-strain plot, and Halder-Wagner methods indicated peak broadening attributable to lattice strain and stacking faults. Optical measurements indicated a reduced band gap of approximately 4.48 eV compared to bulk MgO, attributed to surface states, oxygen vacancies, and quantum confinement effects. The Urbach energy (approximately 168 meV) enhanced the identification of defect-related localized states within the band gap. AC conductivity increased with frequency, supporting Jonscher’s universal power law, whereas impedance spectroscopy revealed grain-dominated dielectric relaxation exhibiting non-Debye behavior in the Cole–Cole plot, characterized by a single semicircular arc. Frequency-dependent dielectric constant and loss demonstrated interfacial and dipolar polarization. This study is the inaugural investigation to directly associate microstructural defects and strain from the synthesis process with the optical and electronic transport properties of the material, demonstrating the sol–gel method’s capability for fabricating functional ceramics with customized attributes for advanced electronic and optoelectronic applications.

**Supplementary Information:**

The online version contains supplementary material available at 10.1038/s41598-026-44397-5.

## Introduction

Nanoparticles, materials with at least one dimension on the nanometer scale (1 − 100 nm), have captured significant scientific and technological interest due to their unique properties that often differ substantially from those of their bulk counterparts^1–6^. This change in behavior at the nanoscale is governed by quantum confinement effects and a dramatic increase in the surface-to-volume ratio^7–12^, which collectively influence their physical, chemical, and electronic characteristics. These novel properties have opened up a vast array of possibilities across multiple fields. In the realm of electronics, for instance, nanomaterials are integral to developing next-generation devices with enhanced performance^13,14^. Their application extends to catalysis and sensing applications^15–19^, where the high surface area of nanoparticles can significantly improve reaction efficiency, and in energy storage, where they enable the creation of more efficient batteries and super capacitors^20,21^.

Among the various materials being explored at the nanoscale, magnesium oxide (MgO) stands out as a promising candidate due to its exceptional properties^21–28^. As a ceramic, MgO possesses a wide band gap of approximately 7.8 eV, which makes it an excellent insulator and a key component in microelectronic devices^29–35^. Its high thermal stability and robust chemical inertness make it suitable for applications in harsh environments, such as high-temperature catalysts and refractory materials^36–39^.

Furthermore, the material’s low electrical conductivity and high dielectric constant position it as a valuable asset for insulating layers and dielectric components in advanced electronic systems^40–42^. However, the properties of MgO nanoparticles are not just a simple extension of their bulk properties; they are heavily influenced by subtle changes in synthesis conditions. These changes can lead to the formation of structural and microstructural defects, such as lattice strain and oxygen vacancies^43,44^. A thorough understanding of these defects is crucial, as they can significantly alter the material’s optical and electronic behavior, offering a pathway to tune its functionality for specific applications.

A comprehensive investigation into how these defects correlate with the transport properties of the material is essential for unlocking its full potential. The remarkable properties of MgO nanoparticles have led to their widespread application across a diverse range of fields, establishing their relevance as a versatile and functional nanomaterial. In catalysis, MgO’s high surface area and basic sites make it an effective catalyst and support for various chemical reactions, including the synthesis of fine chemicals and the remediation of hazardous industrial waste^45,46^. Its utility in electronics is equally significant, where its low electrical conductivity and high thermal stability are exploited in fabricating insulating layers, memory devices, and other components for microelectronic systems.

Furthermore^47,48^ MgO nanoparticles have gained considerable attention in the biomedical field. Their demonstrated antimicrobial properties, which make them effective against a wide range of bacteria and fungi, have led to their use in wound dressings and medical coatings. Researchers are also exploring their potential in targeted drug delivery and as anticancer agents due to their unique interactions with biological systems^24,49,50^. The broad and impactful applications across these domains underscore the critical need for a deeper understanding of how the synthesis and fundamental properties of MgO can be precisely controlled to meet the demands of advanced technological and biomedical applications.

The synthesis of high-purity, homogeneous nanoparticles with controlled size and morphology is critical for their functional application. In this study, magnesium oxide (MgO) nanoparticles were synthesized using the sol–gel method, a technique renowned for its ability to overcome the limitations of traditional synthesis routes. The method, a Sol–gel process, is a robust and versatile approach that ensures excellent chemical homogeneity at the atomic level, which is a key requirement for producing high-quality metal oxide powders^51–53^. On the other hand the sol–gel method has some limitations. A major drawback is the necessity for lengthy drying and processing times, which can be critical since the removal of solvents and organic templates is slow and energy-intensive. Furthermore, the inherent capillary forces generated during the drying process often led to significant shrinkage and cracking of the final monolithic gel, making it difficult to prepare large-scale, crack-free materials. Finally, the final properties of the synthesized material are highly sensitive to processing conditions like pH, temperature, and precursor concentration, requiring precise and careful control to ensure consistent and reproducible results^54,55^.

The fundamental principle of the sol–gel method centers on the controlled chemical reaction of a precursor solution, typically a metal alkoxide or an inorganic salt. This process begins with the formation of a colloidal suspension, or sol, as the precursor undergoes hydrolysis. As the reaction progresses, a subsequent polycondensation step occurs, which causes the nanoparticles within the sol to link together and form a continuous, three-dimensional network known as a gel. The metal cations are then homogeneously dispersed and immobilized within this rigid gel matrix, ensuring their uniform distribution throughout the material. This controlled process is crucial for achieving high purity and homogeneity in the final product^56,57^. An important phenomenon that can be considered a result of the chemical route, such as Sol–gel, is the Schottky defects. The formation of Schottky defects is an expected and inherent result of the sol–gel synthesis route, particularly for metal oxide nanoparticles like MgO. The sol–gel process involves the transition from a liquid sol to a solid gel network, followed by a critical calcination at high temperatures. During this high-temperature treatment, the thermodynamic driving force to create defects is high. To achieve the most stable crystal lattice configuration under these non-equilibrium, high-energy conditions, the material tends to maximize its entropy by generating point defects. Furthermore, the sol–gel method produces material with an extremely high surface-to-volume ratio and many grain boundaries. These boundaries act as easy sinks and sources for vacancies, lowering the overall energy required for the formation of a Schottky defect pair (a magnesium vacancy and an oxygen vacancy). Therefore, the specific thermal processing of the sol–gel derived gel is precisely what leads to the creation and stabilization of the high concentration of Schottky defects that your rigorous XRD analysis confirmed via the non-stoichiometric atomic occupancies^58–60^.

The primary research gap this work addresses is the importance of, quantitative investigation linking the process-induced structural imperfections of sol–gel MgO to its functional performance. Specifically, while MgO is widely studied, previous reports fail to quantitatively correlate the Schottky defects and lattice strain rigorously confirmed by advanced XRD analysis, with the resulting significant optical band gap narrowing and the specific grain-dominated electronic transport mechanism observed in the dielectric properties. Our study is necessary to close this gap by providing a complete, mechanistic feedback loop from synthesis-induced structural defect engineering to final electronic and optoelectronic function.

The motivation for selecting Magnesium Oxide (MgO) and the sol–gel method is driven by the goal of defect engineering and its impact on material functionality. We chose MgO due to its intrinsic wide-bandgap properties and, crucially, its susceptibility to forming Schottky vacancies under high-temperature synthesis. By using the sol–gel route, we aimed to systematically induce and quantify these structural defects. The core motivation is to demonstrate how this controlled non-stoichiometry transforms the material, leading to desirable functional properties, specifically the reduced optical band-gap energy and enhanced dielectric relaxation, which are highly relevant for next-generation electronic and optoelectronic applications. The primary aim of this research is to synthesize high-quality magnesium oxide (MgO) nanoparticles and to establish a direct correlation between their structural properties and electronic transport behavior. This study employs the sol–gel method, a robust wet chemical synthesis technique, to produce the nanoparticles, which are then comprehensively analyzed using a suite of characterization techniques.

## Experimental techniques

**Fig. 1:**
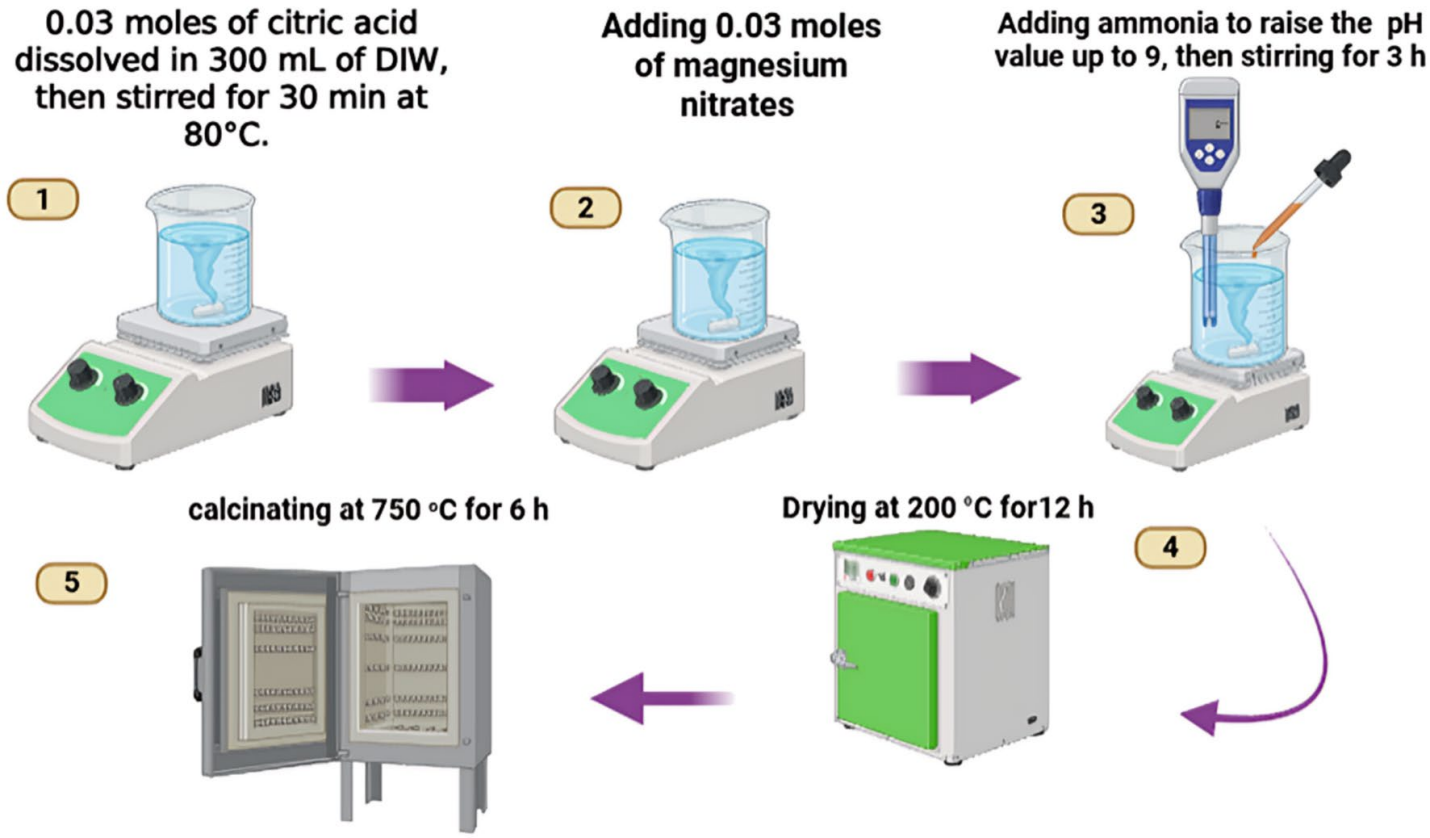
The reaction flowchart for MgO synthesis.

MgO nanoparticles were synthesized using the Sol–gel method. 0.03 mol of citric acid (purchased from Piochem, Purity 99%) were added to 300 ml of De-ionized water (DIW). Then, stirring was increased from 1000 Rpm for 30 min at 80 °C in atmosphere. Then, 0.03 mol of magnesium nitrates Hexahydrated (Mg (NO_3_)_2_.6H_2_O) was added to the solution, leaving the reaction under stirring for 3 h and raising the pH value using ammonia solution until reaching 9. After that, the solution was dried at 200 °C for 12 h in a drier to form a black ash. This ash was later calcined in a muffle furnace in the atmosphere at 750 °C for 4 h. It’s known from the literature that the calcination temperature for MgO nanoparticles synthesized with the (400–900 °C), 750 °C has been chosen as an intermediate temperature, as it’s above 400 °C to conserve crystallinity and below 900 °C to conserve nanostructure^61–63^. Finally, a white powder was formed, ready for analysis and characterization. Figure 1 demonstrates the reaction flowchart.

### Reaction flow chart

## Result and discussion

### Phase analysis

#### Structural study

**Fig. 2:**
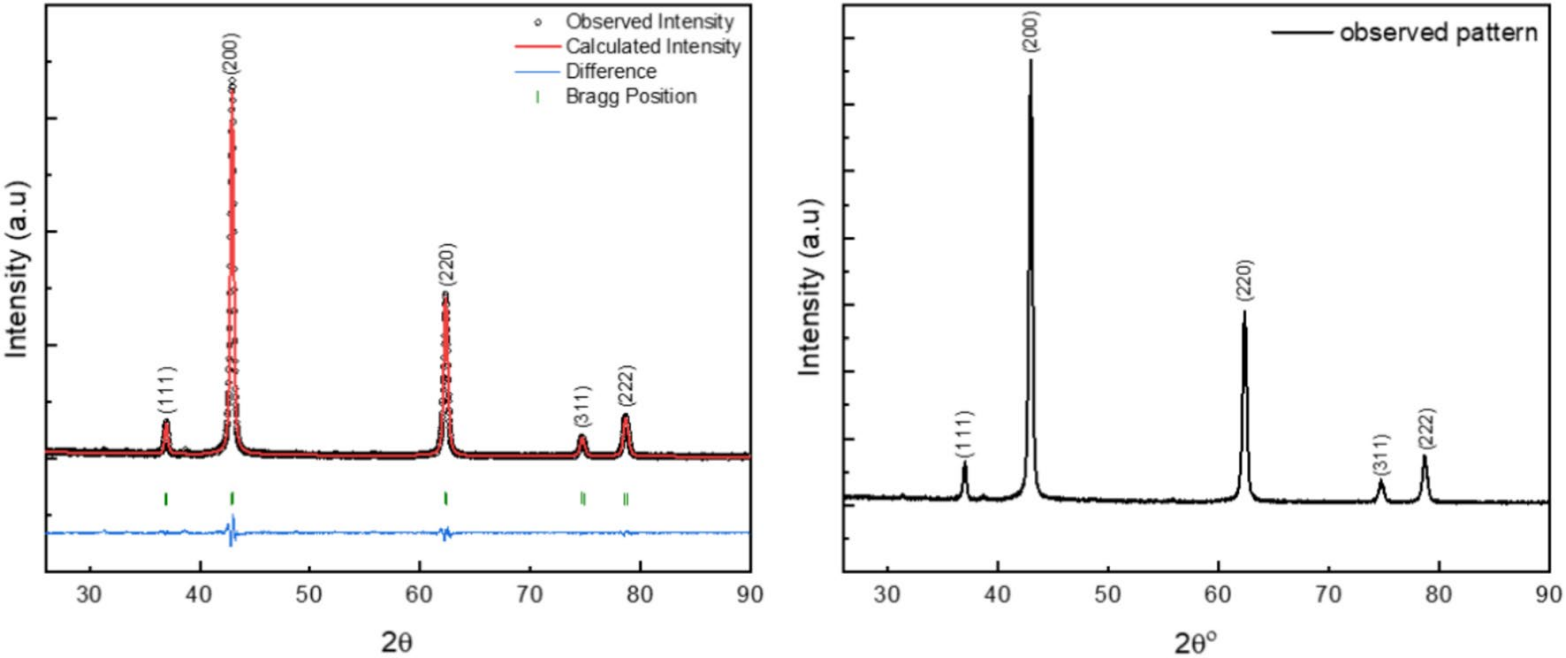
The scanned and refined pattern of MgO nanoparticles.

The XRD method was employed to investigate the reflection pattern for the synthesized MgO. We used the Bragg–Brentano technique to record the reflection pattern. The pattern was recorded with a 25-degree starting angle, 90-degree ending angle, and a step equal to 0.02°. The final pattern was matched using the X’pert high score software from the Malvern Panalytical package. The matching results agreed with the inorganic crystal structures database (ICSD) card number (060692). Rietveld refinement was carried out to confirm phase purity and estimate the most accurate lattice parameters. Full-width at half maximum (FWHM) values have also been deduced from the refinement output, as they will be used in the microstructure calculations. Figure 2 indicates the scanned and refined pattern for the MgO nanoparticles.

Figure 2 exhibits the high crystallinity of the MgO NPs, as the ratio between the background noise and the intensity of the peaks is very small. The pattern has only five peaks, which normally represent the main phase in a cubic structure that appears for the highly crystalline MgO compound^64,65^. The first peaks directed to the (111) direction appeared at 2θ = 36.8°, the second peak directed to the (200) direction appeared at 2θ = 42.8°, the third peaks directed to the (220) direction appeared at 2θ = 62.3°, the fourth peak directed to the (311) direction appeared at 2θ = 74.68° and finally the fifth and the last peak directed to the (222) direction which appeared at 2θ = 78.65°. The obtained results are matched with the published ones^65^.

From Table 1 the goodness of fit obtained from the refinement agrees with the refined pattern. The Rietveld refinement utilizes the Pseudo-Voigt function, which serves as the line broadening β(2θ) function. This function primarily illustrates a peak that amalgamates two profiles: Lorentzian and Gaussian. For the Pseudo-Voigt function (see Eq. S1)^66–68^. The estimated lattice parameters are in agreement with the published ones^69,70^. For the R-factors, also known as Residual factors, sometimes referred to as reliability factors, are mathematical indicators utilized in Rietveld refinement to assess the degree of fit between the estimated pattern derived from the structural model and the observed diffraction pattern^71^. These factors are utilized to assess the degree to which the enhanced model aligns with the experimental results. In Rietveld refinement, the predominant R-factors utilized are: Rₚ (Profile R-factor), R_wp_ (Weighted Profile R-factor), and Rₑₓₚ (Expected R-factor) (See Eq. S2-S4)^72^.

**Table 1:** The obtained lattice parameters and goodness of fitting parameters.

Crystal system	Space group	Lattice parameters	Unit cell volume	Theoretical density	R-factors	Goodness of fitting
Cubic	F m -3 m	a = b = c = 4.2134 (Å) α = β = γ = 90^o^	74.7973 (Å^3^)	3.746 g/cm^3^	R_p_: 17 R_wp_:16 R_exp_: 12.71	χ^2^ = 1.6

The final and most critical criterion is χ^2^ (Goodness-of-Fit, GOF) (See Eq. S5). Also, the Table 1 gives the theoretically calculated density of MgO NPs, which is 3.740 g/cm^3^. The value is larger than the experimental value, as the experimental value is about 3.58 g/cm^3^. The geometrical parameters, represented in the atomic positions, thermal factors, and occupancies, are estimated using the Rietveld refinement.

In Table 2, we notice the atomic positions x, y, and z; these parameters determine the periodic arrangement of atoms inside the lattice, forming a basis of crystallography. Also, these parameters dictate the interatomic distance and bond angles; they are very useful in understanding the electronic and mechanical properties. The thermal factor is a very important quantity. Inside the unit cell, the atoms vibrate around their equilibrium positions. The thermal factor represents the amplitude of the vibration. Here, we have two types of thermal factors, isotropic and anisotropic thermal factors (See Eq. S6)^73^.

**Table 2:** The geometrical parameters, the atomic positions, thermal factors and occupancies estimated using the Rietveld refinement.

Atom	x	Y	z	Thermal factor	Occupancy
Mg	0.000	0.000	0.000	0.28051	0.89858
O	0.500	0.500	0.500	1.07602	0.99345

In the anisotropic one, the vibration around the equilibrium position for the atom is not equal in the whole directions. In this case, it is described by a symmetric 3 × 3 tensor (B_ij_) (See Eq. S7).

**Fig. 3:**
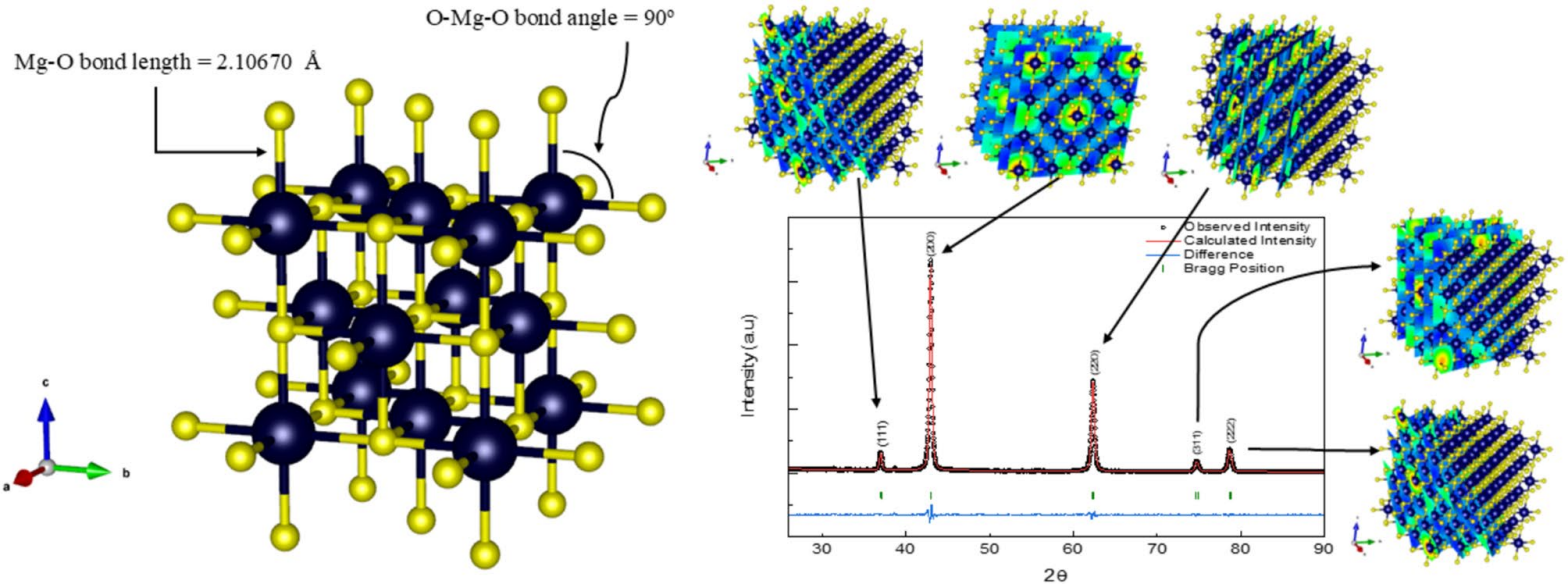
(on the left) The simulated unit cell and the calculated bond length and bond angle, (on the right) a supercell model for relating the peaks in the XRD refined pattern and the simulated atomic planes.

Our case in MgO is described by an isotropic thermal factor, 0.28051 for the Mg atom and 1.07602 for the Oxygen atom. Finally, the atomic occupancy refers to a specific atomic site in a crystal lattice that is occupied by an atom. By another meaning, it is a measure of site filling and is crucial for understanding defects, disorder, and non-ideal stoichiometry in crystals. In cubic MgO (Magnesium Oxide, rock salt structure), the atomic occupancies of Mg and O are typically 1.0 (fully occupied) under ideal conditions. But in the case of high temperature annealing, such as our case, Schottky defects can appear (paired Mg and O vacancies)^74–77^. Schottky defects are one of the most fundamental point defects in ionic crystals, such as MgO, NaCl, and other alkali halides. They consist of paired cation and anion vacancies^59,78^. Small fractions of Mg or O sites may be empty (Occ < 1.0). We saw this for the magnesium and Oxygen, as the result of the refinement confirms that the occupancy for the two atoms is less than one. Another study we have done in our work is a simulation of the crystal structure. We did this using Vesta software^79,80^. Figure 3 illustrates the simulated unit cell for the face-centered cubic MgO.

Figure 3, on the left, illustrates the simulated unit cell for MgO. As seen, the calculated bond length between Mg and O was found to be 2.1067 Å. While the bond angle (O-Mg-O) is found to be 90°. On the other hand, the right figures indicate every peak and its direction inside the unit cell. Those are the planes with which the X-ray is supposed to interact according to Bragg’s law, while the spaces between those planes are the D-spacing values.

### Electron distribution density mapping study (EDDM)

The electron distribution density inside the unit cell has been computed using the G-Fourier tool included in the Fullprof package. In crystallography, electron density mapping provides a three-dimensional representation of the electron density distribution within a crystal. This map is crucial for understanding the precise atomic arrangement, since it indicates the concentration of electrons, which corresponds to the positions of atoms and bonds inside the crystal structure. The structural factors undergo a Fourier transform to get the electron density map. The diffraction data (in reciprocal space) is mathematically translated to yield a representation of the electron density in real space. The electron density ρ(x,y,z) at a place (x,y,z) within the unit cell can be computed mathematically (See Eq. S8)^81–83^.

**Fig. 4:**
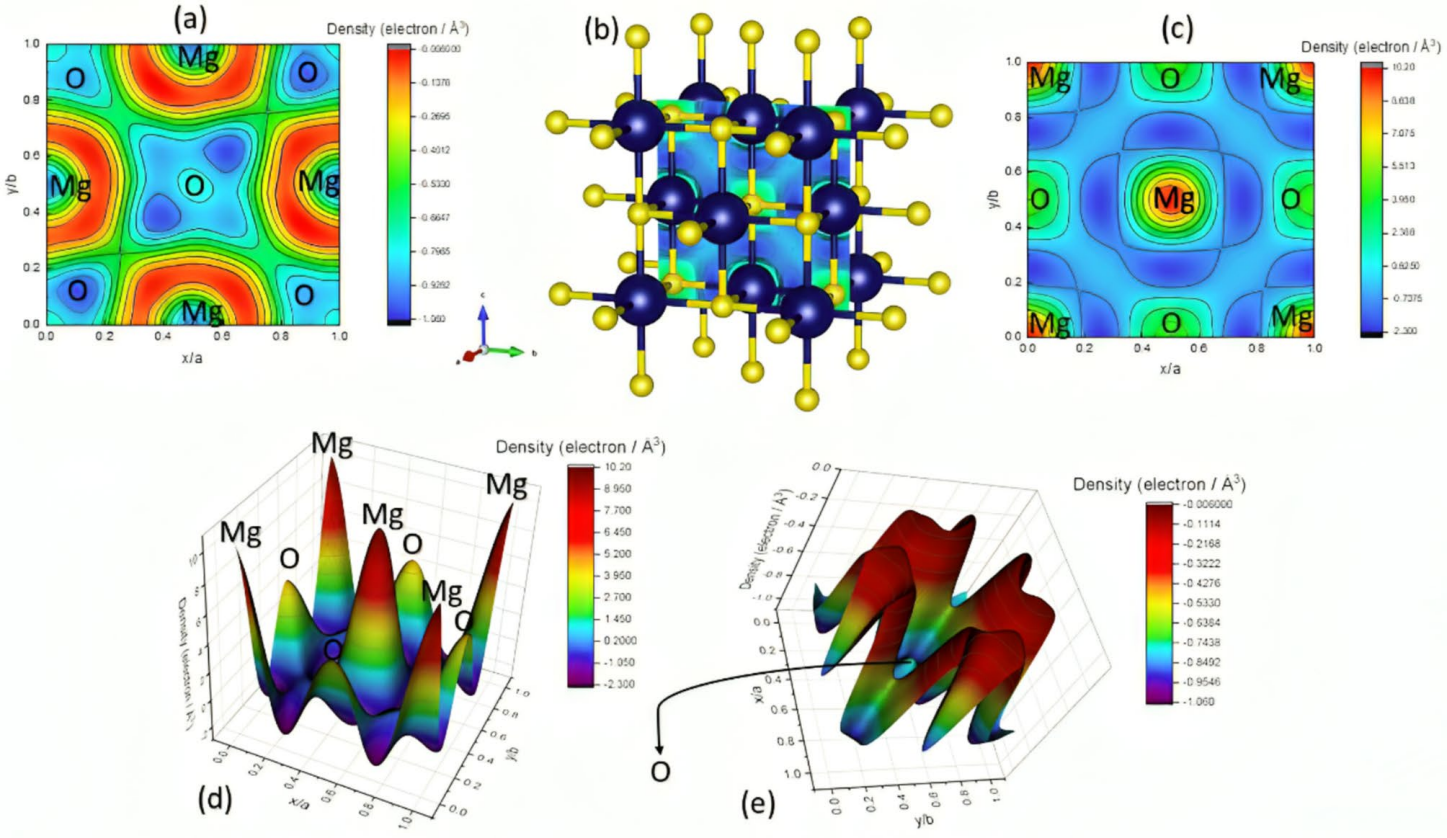
The EDDM for the MgO lattice, (**a**) a contour plot for the EDDM in the (200) direction, (**b**) the face centre cubic unit cell involved with a contour plot for the EDDM in the (200) direction, (**c**) the contour EDDM plot for the (100) direction, (**d**) the 3D visualization plotting for the EDDM in the (100) direction and (**e**) The 3D visualization plotting for the EDDM in the (200) direction.

Electron density mapping reveals that peaks correspond to the positions of atoms within the crystal, as electrons are predominantly concentrated around atomic nuclei and chemical bonds. The atomic number of the constituent elements dictates the intensity of these peaks. Consequently, in comparison to lighter atoms (such as hydrogen or oxygen), heavier atoms (such as metals) manifest as more prominent peaks. Thermal displacement (or Debye–Waller) factors, indicative of atomic motion or disorder inside the crystal, can also be deduced from the shape and distribution of these peaks^84^. Figure 4 illustrates the EDDM for the MgO lattice.

As seen in Fig. 4, the calculated EDDM demonstrates the symmetrical distribution of the electrons among the unit cell, confirming the Face-centered cubic structure for MgO.

We are currently delving further into MgO’s microstructural characteristics. The microstructural characteristics of the MgO have been estimated using the refined full-width at half maximum. These microstructural characteristics are mostly lattice microstrain and crystallite size.^85,86^. Prior to doing those computations, we must take into account that the peak broadening in any material’s XRD pattern carries all of the information regarding its internal flaws. Among those flaws are the lattice microstrain and crystallite size. In theory, the perfect crystal has no broadening, which means that the peak in the XRD pattern does not get wider. In actuality, however, we see broadening in the peaks; this is not the case. We also need to understand that a single crystal and a perfect crystal differ greatly from one another. The material is essentially divided into three major categories based on how crystalline it is. These are amorphous, polycrystalline, and single-crystal materials. The major difference between them lies in the periodic arrangement of atoms. As in the single crystal, there is only one orientation inside the material, which means that you will observe only a single peak in the XRD pattern. This single crystal may be a perfect one or not, depending on the existence of broadening in the peak. Suppose there is no broadening in the peak, so the material is said to be a single and perfect crystal (theoretically, of course). The second type is the polycrystalline material. This material contains many directions or many peaks. Every group of tiny crystals is oriented in a specific direction (Miller indices). Also, this type could be a perfect polycrystalline material if there is no broadening in the peaks. The crystallites in this one are randomly oriented, have no preferred orientation at all; this type is impossible to be a perfect one because, from the beginning, there are no oriented crystallites inside the material^87–89^.

**Fig. 5:**
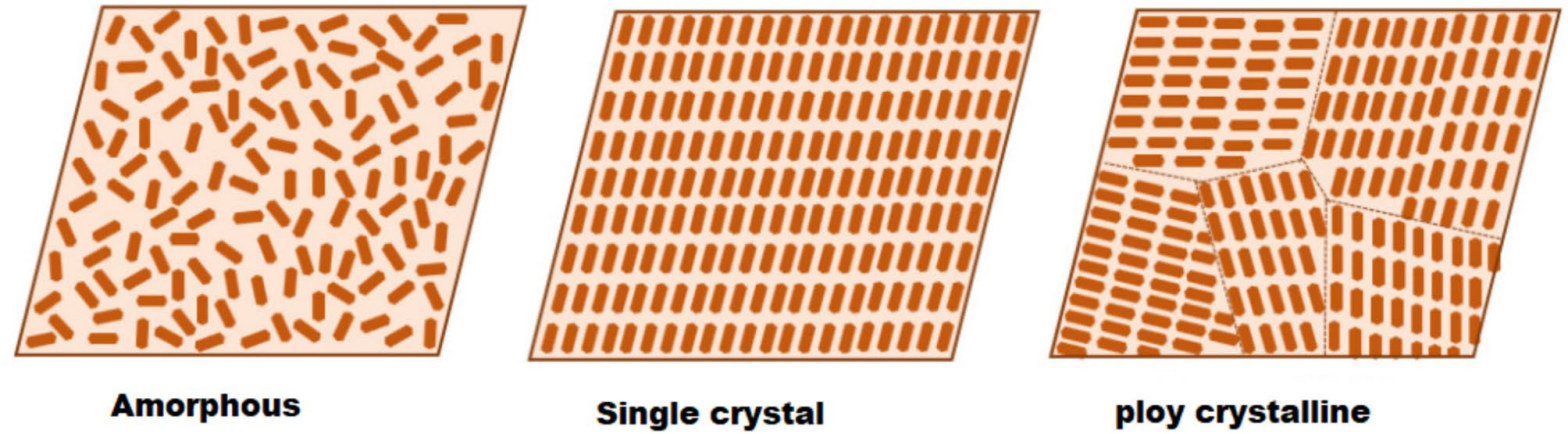
The difference between the single crystals, polycrystalline, and amorphous materials. The image has been cited from the following reference^90^.

Figure 5 illustrates the difference between the three types. Returning to the microstructural analysis, here our material is a polycrystalline material and a non-perfect crystal; besides the crystallite size and lattice microstrain, other defects contribute to the peak broadening, such as the enhancement of the diffractometer. Instrumental broadening (from the diffractometer itself) must be accounted for via calibration with standard samples to isolate the sample-induced effects. Modern diffractometers enhance this process with high-resolution optics, parallel-beam geometries, and advanced software for peak fitting, allowing researchers to distinguish between size and strain effects with high accuracy. This capability is invaluable for applications ranging from nanoparticle synthesis to defect engineering in alloys, where understanding microstructural evolution is crucial for optimizing material performance^91,92^. We will start our computational analysis by subtracting the contribution of the diffractometer from the peak broadening. A standard sample, which is Aluminum oxide (Corundum), is used to calibrate the X-ray diffractometer. This sample has been scanned under the same conditions as the tested sample. After that, Rietveld refinement has been carried out for the reference sample to estimate the resolution profile for the diffractometer. This profile is a mathematical relation between the full width at half maximum (FWHM) and the peak position of the reference sample.

**Fig. 6:**
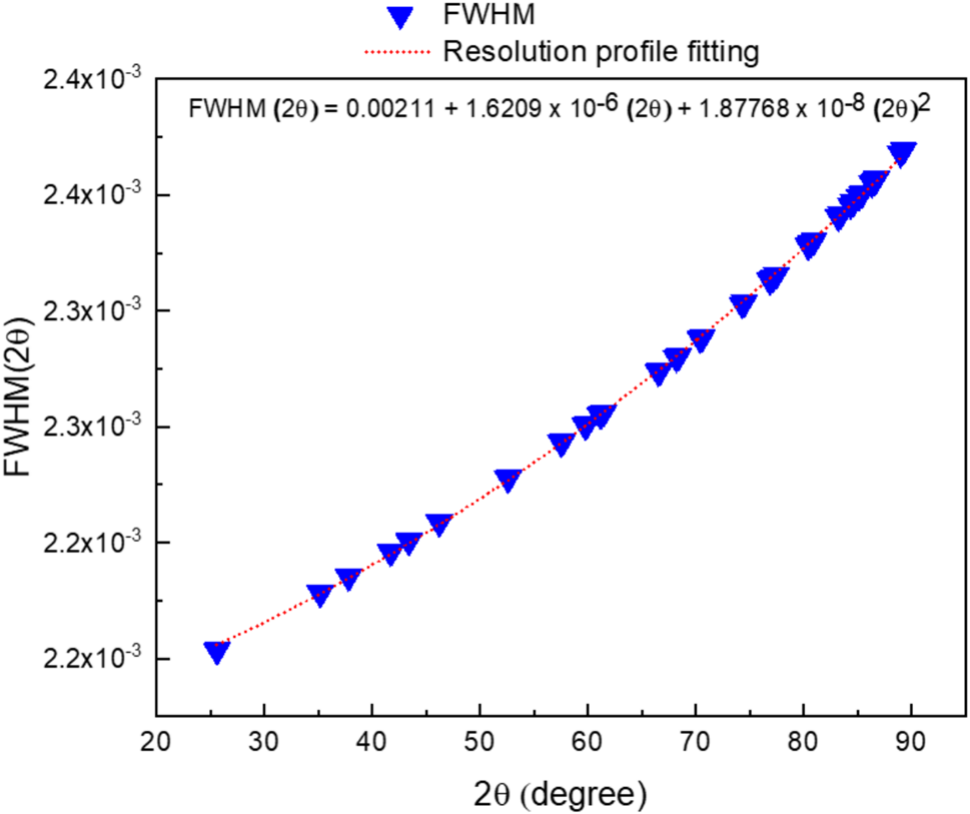
The resolution profile for the used diffractometer.

Figure 6 illustrates the resolution profile for the used diffractometer. The result of the profile fitting is the following equation:

1 $${\mathrm{F}}\left( {{2}\theta } \right) = 0.00{21} + {162}0{9} \times {1}0^{{ - {6}}} \left( {{2}\theta } \right) + {1}.{87768} \times {1}0^{{ - {8}}} \left( {{2}\theta } \right)^{{2}}$$

Important considerations must be taken into account. This equation is not inherent; rather, it serves as a calibration for the XRD equipment utilized in sample testing, as the resolution behaviour may vary over the diffractometer’s lifespan. Subsequently, recalibration will be necessary. Secondly, the reference sample is not only corundum; there are other forms of calibration samples, including lithium hexaborate (LiBO_6_) or a silicon crystal. Regardless of the reference sample, you must create a resolution profile for it. In the next step, we will substitute with the values of the peak positions obtained from the XRD pattern of MgO in the resolution profile equation. The obtained values after substitution are the values contributing to the peak broadening of the MgO XRD pattern. Those values will be eliminated from the refined FWHM values of the MgO using the following equation^68,82,93–95^:

2 $$FWHM_{{corrected}} = \sqrt {FWHM_{{sample}}^{2} - FWHM_{{diffractometer}}^{2} }$$

where *FWHM*_*Corrected*_ are the corrected values used in microstructural calculations, ${FWHM}_{sample }^{2}$ are the refined values of FWHM obtained from Rietveld refinement, and finally ${FWHM}_{diffractometer }^{2}$ the contribution of the diffractometer to the peak broadening. The following table contains the FWHM_Corrected_, FWHM_sample_, FWHM_diffractometer_, peak position, D-spacing, and (hkl) (Table 3).

**Table 3:** The results of hkl, peak position, D-spacing, FWHM_sample_(rad), FWHM_diffractometer_ and FWHM_Corrected_.

Index	hkl	Peak position	D-spacing	FWHM_sample_(rad)	FWHM_diffractometer_	FWHM_Corrected_
1	111	18.4605	2.432562	0.005805872663	0.002146321571	0.005394577008
2	200	21.447	2.10666	0.005721817607	0.002153400279	0.005301137988
3	220	31.1375	1.489634	0.006295978571	0.002178675706	0.005907005869
4	311	37.326	1.270364	0.007445906202	0.002196662120	0.007114505935
5	222	39.2945	1.216281	0.007948753012	0.002202684918	0.007637463820

The predominant microstructural factors are crystallite size and lattice strain. Assuming there is no tension in the lattice. We will commence our computation utilizing the modified Scherrer equation to ascertain the characteristics of our sample. The modified Scherrer equation is predicated on the necessity to minimize errors in the average FWHM values^96,97^. The average crystallite size, derived from the augmentation of all peaks or a selected number of peaks from the reflection pattern, was calculated using the least squares method to minimize mistakes. By applying the natural logarithm to both sides, we get the conventional Scherrer equation^98^:

3 $$D = K\lambda /\beta Cos\left( \theta \right)$$

Applying the natural logarithm to both sides;

4 $$Ln\left| \beta \right| = Lin\left| {\frac{k\lambda }{{D\cos (\theta )}}} \right| = Lin\left| {\frac{k\lambda }{D}} \right| + Ln\left| {\frac{1}{{{\mathrm{Cos}} (\theta )}}} \right|$$

where β is the corrected FWHM value, D is the crystallite size, k is the shape factor, usually taken as 0.9, and λ is the intrinsic wavelength of CuKα_1_ target, which is 1.5406 Å. The slope of the plot of Ln|β| vs Ln|1/Cos(θ)| must equal one. Nevertheless, the least squares method produces the optimal slope and the most precise intercept Ln |kλ/D| due to the imperfections inherent in experimental data. The crystallite value might be estimated from the intercept. A feature of the modified Scherrer equation is its ability to minimize the sum of the absolute values of errors $\sum {(\pm Ln\left|\beta \right|)}^{2})$. A single line is drawn through the points, resulting in a singular intercept value Ln|Kλ/D|. A piece of important information must be taken into consideration, which is the wavelength we use in angstroms, so the obtained size will be in angstroms, then it will be converted to nanometres.

**Fig. 7:**
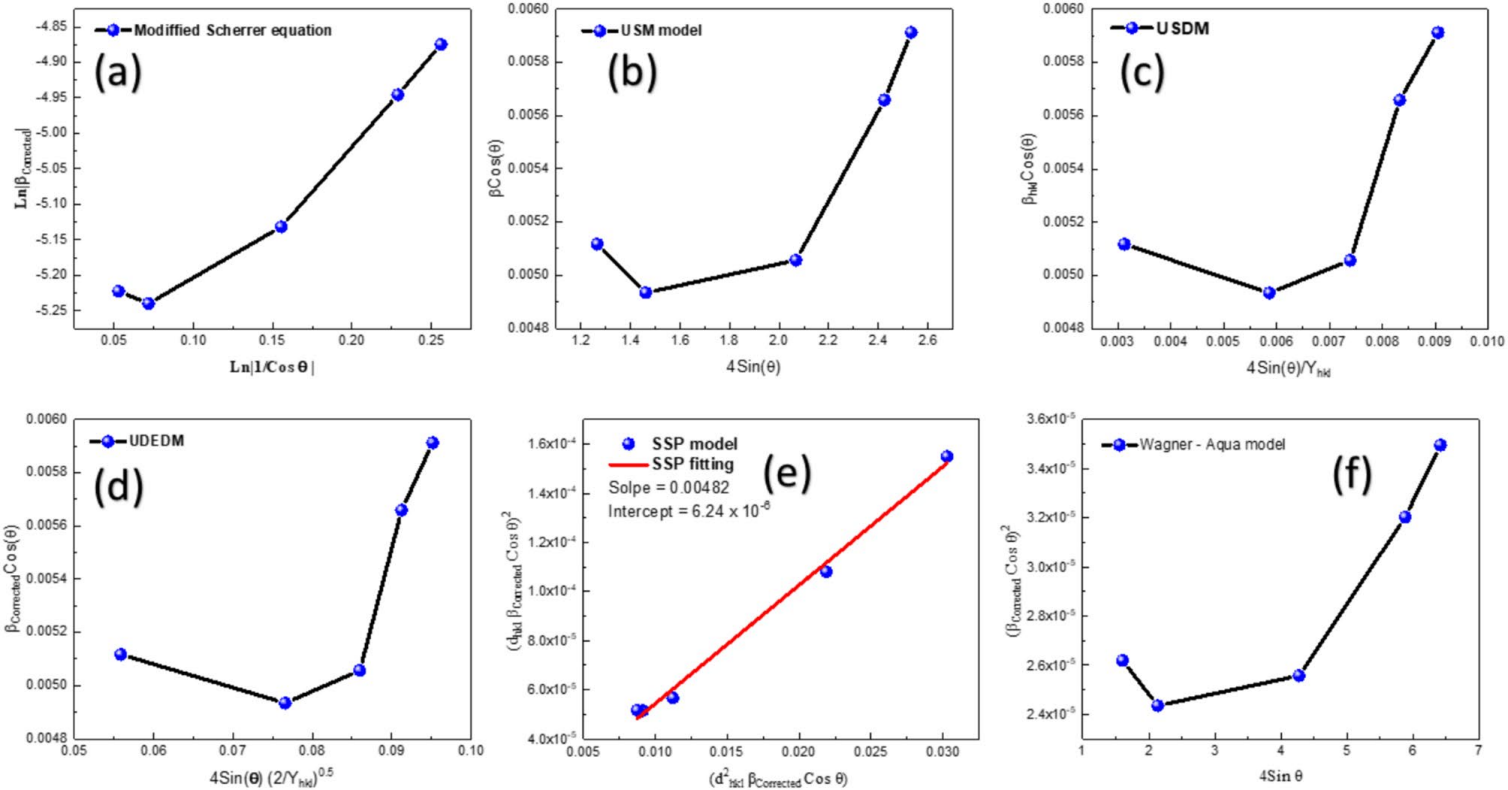
(**a**):The results derived from the modified Scherrer equation, (**b**): USM, (**c**) USDM, (**d**) UDEDM, (**e**) SSP, and (**f**) Wagner-Aqua.

From Fig. 7a, it seems that the data have deviated from linearity; the reason behind this is the existence of strain between the crystallites. To obtain the value of this strain, we will apply the Williamson-Hall techniques^99^. Those techniques are three, the first one is the uniform strain model (USM) with indicated an average value of strain in the entire sample, in another terms, the Strain is said to be isotropic, the second one is the uniform stress deformation model (USDM) which indicates that the strain is not equalled in the whole direction inside the material, instead of that every direction (peak) has its strain and thus can be calculated using the Hook’s law using and average value of stress computed from this model. Finally, the third model is the uniform deformation energy density model (UDEDM). This model states that the energy exerted between the lattice planes is uniform. We will apply the three models for MgO. Starting with the (USM) model, the Williamson-Hall approach asserts that the total complete width at half maximum reflects the crystallite size while considering the influence of strain on peak broadening^100–104^.

The strains arise from several factors, including point defects, grain boundaries, triple junctions, and stacking faults^105,106^. The Williamson-Hall hypothesis, predicated on microstrain, associates the Gaussian component, on the other hand, with crystallite size, which is ascribed to the Lorentzian component. The subsequent equation delineates the overall broadening attributable to strain and crystallite dimensions.

5 $$\beta_{sample} = \beta_{crystallite \, size} + \beta_{strain}$$

where β_sample_ represents the entire broadening after correction, *β*_*crystallite size*_ denotes the broadening attributed to crystallite size, and β_strain_ signifies the broadening resulting from microstrain. The strain value can be articulated as follows^107,108^:

6 $$\beta_{strain} = 4 < \varepsilon > tan\left( \theta \right)$$

The root mean square of the strain is represented by < Ɛ > ; the broadening attributed to crystallite size will be assessed using the Scherrer equation, so the Williamson-Hall equation for the (USM) model will be:

7 $$\beta _{{sample}} cos(\theta ) = \frac{{K\lambda }}{D} + 4 < \varepsilon > sin(\theta )$$

by plotting *β*_*sample*_* cos(θ)* on the y-axis and *4 sin(θ)* on the x-axis, the slope is < *Ɛ* > *,* and the crystallite size is obtained from the intercept.

Figure 7b demonstrates the application of the USM model for the MgO sample; it is clear that there is also a deviation from linearity, which means that the strain is not the same in all directions. Every peak contains a group of crystallites that are oriented in a specific direction; those crystallites corresponding to every peak have their own strain, which means that the crystallites’ microstrain is anisotropic in nature. The non-linearity observed in a Williamson-Hall (W–H) plot using the standard Uniform strain Model (USM) arises primarily because this model fails to account for two critical real-world material characteristics. The foremost reason is anisotropic strain, as we mentioned before, the UDM incorrectly assumes that microstrain is uniform across all crystallographic directions, but in reality, the strain varies significantly between different (hkl) reflections. This variation is due to the material’s elastic anisotropy (the stiffness, or Young’s modulus, which changes with direction) and the directional nature of dislocation strain fields. The second major cause is the inaccurate peak profile assumption. The USM’s linear additivity (*β*_*sample*_ = *β*_*crystallite size*_ + *β*_*strain*_) _*is*_ only mathematically rigorous if both size and strain components are purely Lorentzian, whereas real peaks are typically Voigtian (a convolution of Lorentzian and Gaussian profiles). Since crystallite size broadening is often Lorentzian-like and strain broadening is Gaussian-like, their widths are not strictly linearly additive, causing systematic deviation from the ideal straight line. To overcome this dilemma, we will try the USDM to calculate the average value of stress exerted on the material. And then we will use it to estimate the anisotropic strain. Starting from Hook’s law, *E*_*hkl*_ = *Ɛ Y*_*hkl*_. The equation of the (USDM) will be:

8 $$\beta _{{corrected}} cos(\theta ) = \frac{{K\lambda }}{D} + 4\frac{{E_{{hkl}} }}{{Y_{{hkl}} }}sin(\theta )$$

where *Y*_*hkl*_ is the Young’s modulus, for a cubic unit cell, its value can be estimated from the following equation^109,110^:

9 $$\frac{1}{{\mathrm{Y}}_{\mathrm{hkl}}}=\mathrm{S}11+2(\mathrm{S}11-\mathrm{S}12- \frac{1}{2}\mathrm{S}44)({\mathrm{m}}_{1}^{2}{\mathrm{m}}_{2}^{2}+{\mathrm{m}}_{2}^{2}{\mathrm{m}}_{3}^{2}+{\mathrm{m}}_{3}^{2}{\mathrm{m}}_{1}^{2})$$

where S_ij_ are elastic compliances for cubic MgO, their values are listed in the Table 4.

**Table 4:** The elastic compliances for cubic MgO.

S_11_ = 4.01 TPa^−1^	S_12_ = 6.46 TP^−1^	S_44_ = − 0.96 TPa^−1^	^110,111^

The m’s values are as follows;

10 $$m_{1} = h\left( {h^{2} + k^{2} + l^{2} } \right)^{ - 0.5}$$

11 $$m_{2} = k \, \left( {h^{2} + k^{2} + l^{2} } \right)^{ - 0.5}$$

12 $$m_{3} = l \, \left( {h^{2} + k^{2} + l^{2} } \right)^{ - 0.5}$$

The obtained value of the Young’s modulus for the MgO in whole directions is illustrated in Table 5.

**Table 5:** The obtained value of the Young’s modulus for the MgO in whole directions.

(hkl)	Y_hkl_ GPa^110^
(111)	405.954
(200)	249.377
(220)	279.720
(311)	291.138
(222)	279.720

By substituting in Eq. (16), then plotting *β*_*corrected*_* cos(θ)* on the y-axis and *4Sin(θ)/Y*_*hkl*_, the slope is the stress exerted between the atomic planes, and from the intercept we obtain the crystallite size.

Figure 7c illustrates the USDM model plotting. The deviation from linearity, which has been confirmed in Fig. 9 indicates a non-homogeneous stress exerted between the crystallites. The persistent deviation from linearity in the Uniform Stress Deformation Model (USDM) Williamson-Hall plot, even after correcting for elastic anisotropy using Young’s Modulus, suggests that the actual microstructural conditions are more complex than the USDM’s core assumptions allow. The primary reason for this continued non-linearity is the non-uniformity of the internal stress field, meaning the fundamental hypothesis of constant stress across all crystallographic directions is invalid. This non-uniform stress often arises from highly localized and heterogeneous defect structures, such as complex arrangements of dislocations forming cell walls or dense tangles, where the resulting stress varies significantly from grain to grain. Furthermore, the plot’s scatter may be compounded by the model’s inability to account for dislocation contrast factors, which capture the directional strain effects specific to the Burgers vector of the dominant dislocation type. Finally, the inherent difference in peak profile shapes, where size broadening is often Lorentzian and strain broadening is Gaussian, means that the USDM’s linear summation of widths remains an approximation that is inherently susceptible to error, resulting in persistent non-linear behaviour that neither the UDM nor the USDM can fully resolve.

The final attempt is the uniform deformation energy density model (UDEDM). While the UDM model presupposes the isotropic characteristics of the crystal, the USDM model posits a linear correlation between stress and strain, in accordance with Hooke’s law. However, in actual crystals, the isotropic nature and linear proportionality between stress and strain cannot be assumed, as various flaws, dislocations, and agglomerates introduce imperfections in nearly all crystals. A distinct model is necessary for the examination of various crystal microstructures. The Uniform Deformation Energy Density Model (UDEDM) is employed for this purpose, accounting for uniform anisotropic lattice strain in all crystallographic directions, with the source of this strain being the density of deformation energy^106,112^. With the help of Eq. (9), Hook’s law can be written in terms of deformation energy as follows:

13 $${\mathrm{U}} = \left( {\varepsilon^{{2}} {\mathrm{Y}}_{{{\mathrm{hkl}}}} /{2}} \right)$$

So the strain can be written in terms of energy as follows:

14 $$\upvarepsilon = {\text{E }}\left( {\sqrt {\frac{{2U}}{{Y_{{hkl}} }}} } \right)$$

By substituting into the Eq. (21), the following equation states the UDEDM model;

15 $$\beta _{{corrected}} cos(\theta ) = \frac{{K\lambda }}{D} + 4\sqrt {\frac{{2U}}{{Y_{{hkl}} }}} sin(\theta )$$

Here in, the plot will be between *β*_*corrected*_* cos(θ)* on the y-axis and* 4* $\sqrt{\frac{2}{{Y}_{hkl}}}$ *sin(θ)* on the x-axis, and the slope will be $\sqrt{U}$. The value of U will be very helpful in estimating the value of strain using Eq. (14). Figure 7d illustrates the UDEDM plot for the MgO sample.

Another Dilemma has been observed in the last attempt in the Williamson-Hall family; there is also a deviation from linearity. This indicates that the energy exerted between the lattice planes is not uniform at all, and it also has an anisotropic nature. The Williamson-Hall method examined the combined effects of strain and size-induced broadening on the XRD peak. In contrast, we introduce a size strain model, the (SSP) technique, which emphasizes the analysis of the XRD peak profile, utilizing a combination of a Lorentzian function (β_Lorentzian_) and a Gaussian function (β_Gaussian_) to account for the peak profile. This theory posits that the Lorentz function characterizes the size-broadened XRD profile, while the Gaussian function delineates the strain-broadened profile^113^. Consequently, the SSP approach can be denoted as;

16 $$\beta_{{{\mathrm{Corrected}}}} = \, \beta_{{{\mathrm{Lorentzian}}}} + \, \beta_{{{\mathrm{Gaussian}}}}$$

where β_Lorentzian_ is the peak broadening in the peak, due to the Lorentzian enhancement (size effects), and β_Gaussian_ is the peak broadening in the peak, due to the Gaussian enhancement (strain effects). SSP consistently yields superior results for isotropic broadening because of its focus on low-angle reflections, resulting in enhanced accuracy at broader angles. The quality of XRD data diminishes as diffracting angles increase, resulting in significant peak overlap at higher angles. Consequently, the equation addressed in SSP is as follows:

17 $$({\mathrm{d}}_{{{\mathrm{hkl}}}} \;\beta_{{{\mathrm{Corrected}}}} {\mathrm{Cos}} \;\theta )^{2} = \frac{k\lambda }{D}({\mathrm{d}}^{2}_{{{\mathrm{hkl}}}} \;\beta_{{{\mathrm{Corrected}}}} {\mathrm{Cos}} \;\theta ) + \left( {\frac{ < \varepsilon > }{2}} \right)^{2}$$

where d_hkl_ is the interplanar spacing in Angstroms, β_Corrected_ is the corrected full width at half maximum in radians, k is a shape factor usually taken as 0.95, λ is the intrinsic wavelength of the CuK_α1_ = 1.5406 Å, θ is the angular position in degrees, D is the crystallite size estimated in angstroms, and finally, $<\varepsilon >$ It is the root mean square of the strain. By plotting (d^2^_hkl_ β_Corrected_ Cos θ ) on the x-axis and (d^2^_hkl_ β_Corrected_ Cos θ) on the y-axis, the slope is $\frac{k\lambda }{D}$ and the intercept is ($\frac{<\varepsilon >}{2}$)^2^.

In Fig. 7e, finally, we see a glimmer of hope. The behavior seems to be linear at the end, the estimated slope in this case was 0.00482, corresponding to a crystallite size equal to 30.36 nm, and the intercept was 6.24 × 10^–6^ corresponding to an average root mean square for the strain equal to 0.004995. Now, we are seeking more accurate results. Another attempt has been carried out using a model called (Wagner—Aqua model). Wagner and Aqua provide an additional effective method for estimating lattice strain with crystallite size. All indexed diffraction peaks are incorporated in this method and can be articulated as follows^114^:

18 $$(\beta_{{{\mathrm{Corrected}}}} {\mathrm{Cos}} \theta )^{2} = (4 < \varepsilon > {\mathrm{Sin}} \theta )^{2} + \left( {\frac{k\lambda }{D}} \right)^{2}$$

The terms of this equation are verified in the SPP model, so by directly plotting (β_Corrected_ Cos θ)^2^ on the y-axis and 4Sin θ on the x-axis, the slope is $<\varepsilon >$ and the crystallite size estimated from the intercept ($\frac{k\lambda }{D}$). Figure 7f indicates the application of the Wagner-Aqua model for MgO.

The same dilemma is observed in this attempt, introducing another model to estimate the crystallite size and strain, which is the Halder-Wagner method. This method treats the XRD broadening function as a symmetric Voigt function. Although the XRD profile’s peak region aligns well with the Gaussian function, the peak tail diminishes too swiftly to achieve a proper match. The contrary situation transpired with the Lorentz function, wherein the peak region does not correspond, although the profile tails align satisfactorily^115^. Halder-Wagner proposes a method that modifies the SSP technique to create a relationship between lattice strain and crystallite size. The full width at half maximum of the symmetric Voigt function can be articulated about the Halder-Wagner approach as follows^116^.

19 $$\beta^{{2}}_{{{\mathrm{Corrected}}}} = \beta^{{2}}_{{{\mathrm{Lorentzian}}}} + \beta^{{2}}_{{{\mathrm{gaussian}}}}$$

where, meaning of β_Lorentzian_ and β_Gaussian_ is the same as mentioned before. One advantage of this method is that it places greater emphasis on the peaks at low and medium angles, where there is less overlap between diffracting peaks. Now, based on the Halder-Wagner Method approach, the relationship between lattice strain and crystallite size is provided as follows.

20 $$\left( {\frac{\upbeta }{{{\mathrm{tan}}(\upbeta )}}} \right)2 = \frac{{k\lambda }}{D}\frac{\beta }{{Sin\left( \theta \right){\mathrm{tan}}(\theta )}} + 16 < \varepsilon >$$

**Fig. 8:**
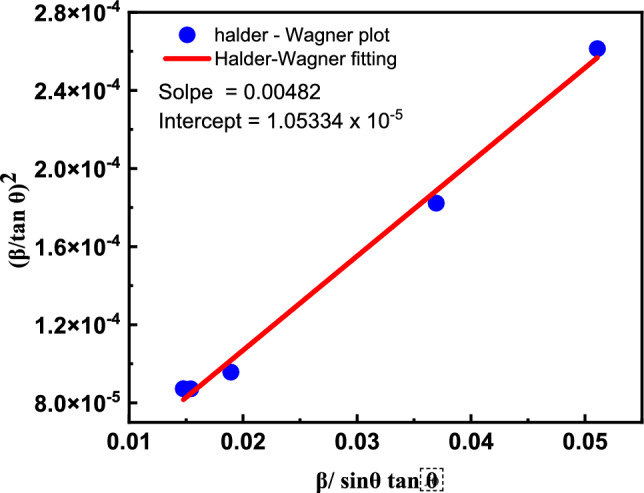
The Halder-Wagner plot for MgO.

Plotting the $(\frac{\upbeta }{\mathrm{tan}(\uptheta )})$^2^ on the y-axis and $\frac{\beta }{Sin\left(\theta \right)\mathrm{tan}(\theta )}$ on the x-axis, the crystallite size could be obtained from the slope, and the strain from the intercept. Figure 8 illustrates the Halder-Wagner plot for the MgO sample.

The results obtained from the Halder-Wagner method are very close to those obtained from the size strain plot, especially in the crystallite size. As the crystallite size in the case of Halder-Wagner was 30.364 nm, while the estimated strain was 0.00649.

Moving to another part, which is the stacking faults, stacking faults are planar imperfections in crystalline materials resulting from errors in the optimal stacking sequence of atomic layers. In close-packed structures, such as face-centered cubic (FCC) or hexagonal close-packed (HCP) lattices, disruptions in the typical ABCABC… or ABAB… stacking order due to local displacements lead to regions exhibiting an alternate stacking sequence. These faults are defined by a displacement vector, typically represented by the Shockley partial dislocation in FCC metals^117,118^, wherein the faulted area assumes an HCP-like shape. The stacking fault energy (SFE), which represents the formation energy of stacking faults, is crucial in influencing material properties such as deformation processes, dislocation mobility, and mechanical behavior. Materials with low stacking fault energy (SFE) typically demonstrate significant stacking fault development, facilitating twinning and diminishing cross-slip, whereas materials with high SFE favor dislocation glide. The stacking fault appears in the contribution to the peak broadening, and its value can be computed using the following equation^68,119^:

21 $$Stacking\;fault(Rad) = \left( {\frac{{2\pi ^{2} }}{{45(3tan\theta )^{{0.5}} }}} \right)\beta$$

**Fig. 9:**
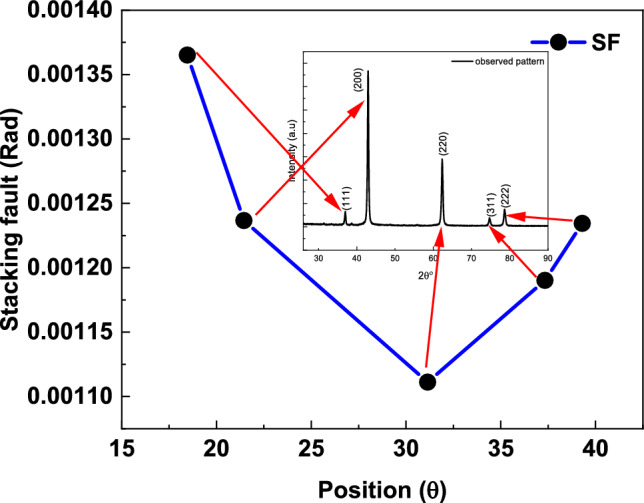
Stacking fault behavior in MgO sample.

Figure 9 indicates the stacking fault behavior in the whole direction in the MgO sample.

From Fig. 9, the stacking fault starts with a maximum value equal to (0.001365) Rad in the (111) direction and then begins to decrease to reach a minimum value equal to (0.0011112) Rad in the (220) direction and then increases again to reach a value equal to (0.001234) Rad. The reason behind this is that the peak is oriented in the direction (220) corresponding to the smallest value of stack fault, which has the smallest FWHM value, and from Eq. (21), the stack fault is a strong function in peak broadening^120,121^.

While the Modified Scherrer (MS) provides a quick estimation and the Williamson-Hall (UDM-WH) plot offers an initial visual separation of size and strain, the practical application to our chemically synthesized MgO nanoparticles revealed that the Williamson-Hall plots exhibited significant deviation from linearity. This scatter confirms the presence of highly anisotropic crystallite domains and heterogeneous strain fields, rendering its simple linear fit unreliable for precise parameter extraction. Consequently, only the Size-Strain Plot (SSP) and the Halder-Wagner (HW) models provided the high degree of linearity necessary to confidently decouple the size and strain components of the observed peak broadening. This superior fit is attributed to the more sophisticated modelling of the peak profiles: the HW model and the SSP assume a Lorentzian crystallite size distribution combined with a Gaussian strain distribution correlation; its derived microstructural parameters (30 nm) and (ε = 0.004995 for the size strain plot and ε = 0.0064 for the Halder—Wagner) are deemed the most accurate and physically meaningful. The consistency of the SSP and HW linearity confirms the rigor of the advanced X-ray Line Profile Analysis approach, providing the validated parameters used for all subsequent discussions on dislocation density and the effect of defects on optical properties.

Unlike studies that use simple Scherrer calculations, our multi-model approach (W–H, SSP, H-W) reveals significant lattice strain. This is higher than in bulk MgO due to “surface pressure” effects, where the lattice constants of nanoparticles contract or expand to minimize surface free energy^122,123^.

#### HR-TEM analysis

**Fig. 10:**
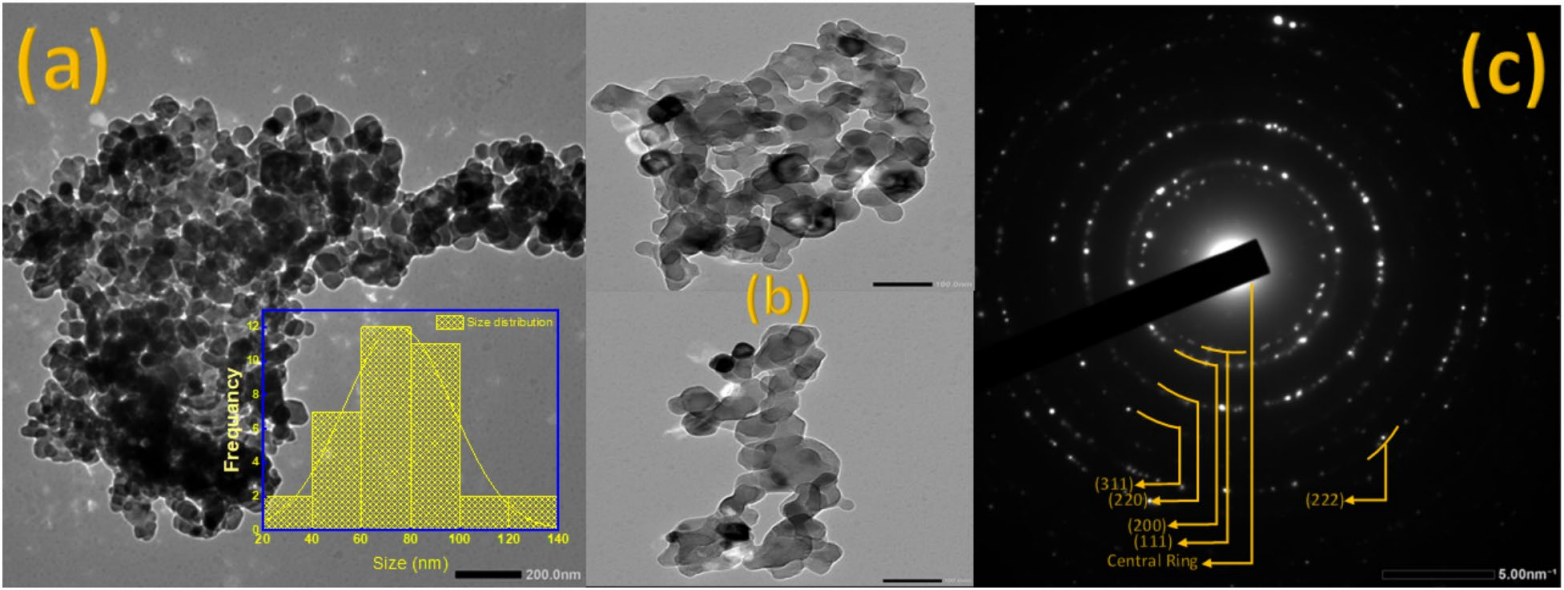
(**a**) HR-TEM image mode for the MgO with a magnification of 200 nm, (**b**) MgO image mode with a magnification of 100 nm, and (**c**) is the selected area electron diffraction pattern for MgO.

High-Resolution Transmission Electron Microscopy (HRTEM) is a critical analytical technique for determining crystallite size with sub-nanometer precision. By directly imaging atomic lattice fringes, HRTEM enables the visualization of individual crystallites and their boundaries, facilitating accurate size measurements through digital image analysis^124,125^. The technique employs Fast Fourier Transform (FFT) patterns to verify crystallographic orientation and periodicity, distinguishing between single-crystalline and polycrystalline domains^126,127^. In our case, MgO has been portrayed at the nanoscale. Transmission electron microscopy (TEM) analysis of the synthesized magnesium oxide (MgO) nanoparticles, synthesized using the sol–gel method, reveals a morphology characterized by the formation of significant agglomerates. The primary particles exhibit a quasi-spherical or irregular polyhedral shape. Based on the 200 nm and 100 nm scale bars from the bright-field image, see Fig. 10a and b, the average size of the individual, primary nanoparticles is estimated to be approximately 30–50 nm. The image shows that these primary particles are not well-dispersed but have formed larger, dense clusters and chain-like structures. This extensive aggregation is a typical characteristic of particles synthesized by the sol–gel route, often attributed to inter-particle bonding during the gelation and subsequent thermal treatments. The average particle size is about 50 nm.

The selected area electron diffraction (SAED) patterns, see Fig. 10(c), obtained from the nanoparticle aggregates, exhibit concentric bright rings composed of discrete, sharp diffraction spots. This characteristic pattern confirms the polycrystalline nature of the synthesized MgO nanoparticles. The presence of distinct spots within the rings, rather than continuous rings, further suggests that the crystallites are well-formed and randomly oriented. The varying diameters of these rings correspond to different crystallographic planes of the MgO structure, indicating good crystallinity throughout the sample.

Here is the Table 6 contains a comparison of the used models to estimate crystallite size and strain, and the results obtained from HR-TEM.

**Table 6:** The obtained results from the used models.

Model property	Modified Scherrer method	Williamson-Hall (USM)	Williamson-Hall (USDM)	Williamson-Hall (UDEDM)	Size-strain plot	Wagner-Aqua	Halder-Wagner	HR-TEM
Crystallite size (nm)	–	–	–	–	30.364 ± 2.09188 × 10^–4^	–	30.364 ± 6.4723 × 10^–6^	Average size (75 nm)
Strain	–	–	–	–	0.004995 ± 3.83711 × 10^–6^	–	0.0064 ± 2.09155 × 10^–4^	–

The previous table gives a comprehensive comparison of the crystallite size obtained from the tested models and the average value obtained from the HR-TEM. Its obvious that the average size obtained from the HR-TEM is larger than the one obtained from Halder–Wagner and the size strain plot, The discrepancy between the crystallite size determined by X-ray Diffraction (XRD) analysis (D = 30.6 nm) and the average particle size observed via High-Resolution Transmission Electron Microscopy (HR-TEM) (D = 75 nm) is a consequence of the fundamental difference in the measured parameters. XRD line-broadening methods (such as Williamson-Hall, Halder-Wagner, and the Size-Strain Plot) measure the size of the Coherently Diffracting Domain (CDD), which represents the extent of the crystal lattice continuity. Conversely, HR-TEM measures the physical particle size, defined by the external boundaries visible in the image. The observation D_XRD_ < D_TEM_ implies that the average 75 nm particle is polycrystalline and is subdivided into smaller crystalline units of 30 nm. This subdivision is governed by the presence of internal lattice defects such as low-angle grain boundaries, high densities of dislocations, and stacking faults, which terminate the coherent scattering of X-rays, thereby restricting the CDD size. Therefore, while HR-TEM accurately measures the particle’s external morphology, XRD provides a volume-weighted average of the internal perfection of the crystal structure, confirming that microstrain and defects are effectively limiting the size of the perfect crystal regions within the larger particles^128,129^.

### Optical properties

**Fig. 11:**
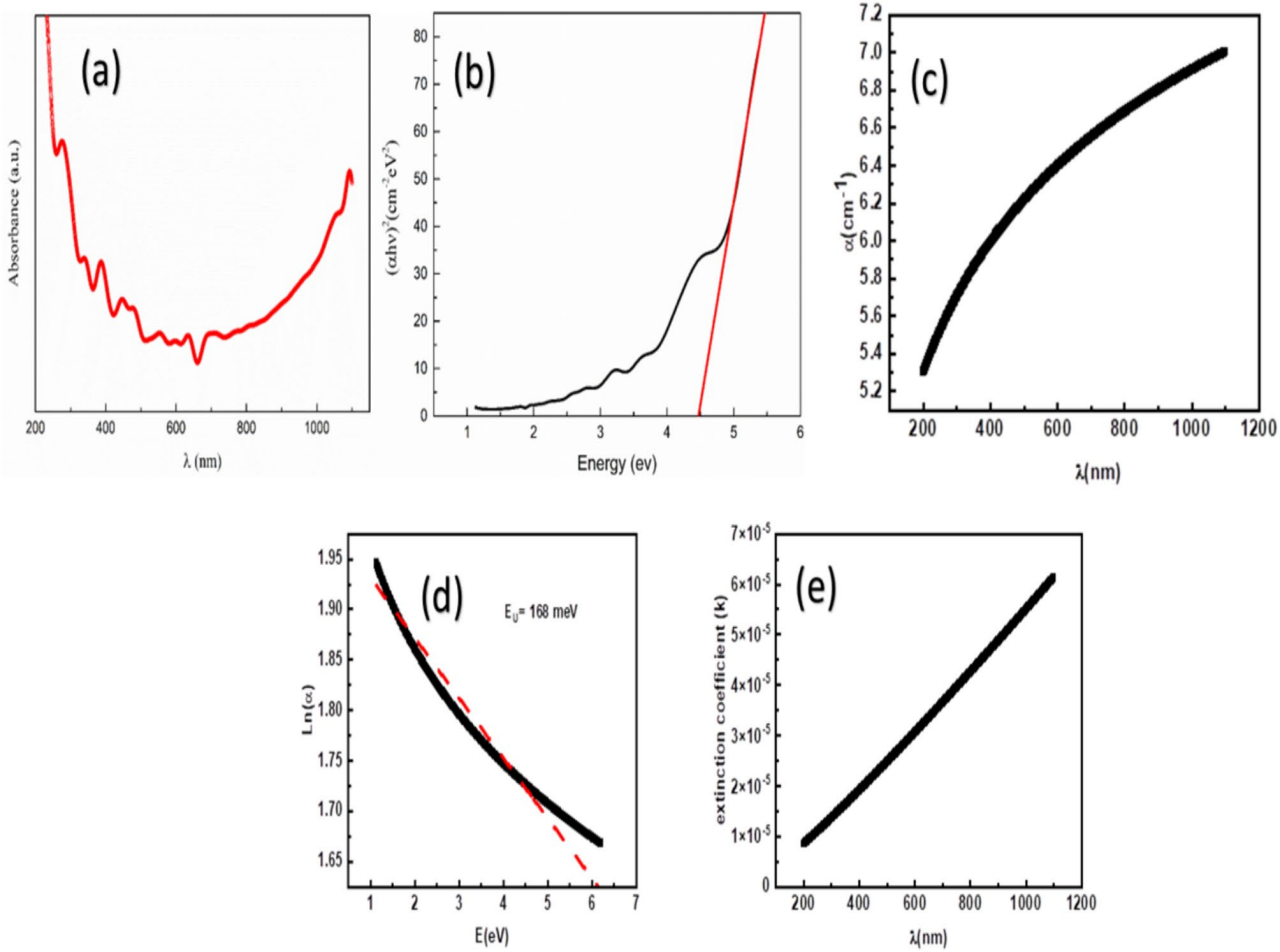
(**a**) The dependence of the absorption on the wavelength studied for MgO nanostructure. (**b**) The dependence of (αhν)^2^ as on the light energy, E(eV), of MgO nanoparticles prepared by the sol–gel method. (**c**) The dependence of the absorption coefficient, α(λ), on the wavelength, λ(nm), studied for MgO nanostructure. (**d**) The dependence of Ln(α), on the light energy, E(eV), studied for MgO nanostructure. (**e**) The extinction coefficient as a function of the light wavelength of MgO nanoparticles prepared by the sol–gel method.

The optical properties of sol–gel-prepared MgO nanoparticles were investigated using UV–Visible to Near-IR (UV-NIR) spectroscopy (200–1100 nm) to study their absorption characteristics and electronic transitions. Figure 11 illustrates the absorption spectrum of the obtained MgO. The absorbance curve exhibits a sharp onset at approximately 276 nm.

The exponent n reflects the transition probability between the valence and conduction bands, where n = 1/2 is for Direct Allowed Transition, which is typically used for MgO, where the valence band maximum and conduction band minimum occur at the same point in the Brillouin zone (Γ-point). While n = 2 is for the Indirect Allowed Transition, which is used when the transition requires phonon assistance to conserve momentum. While we acknowledge that high defect densities and oxygen vacancies can create a distribution of states that mimics indirect-like behavior (due to localized states in the band tail), MgO remains fundamentally a direct band gap material. The best linear fit for our absorption data was achieved using n = 1/2. In literature, even when MgO is nanostructured or doped, the direct transition model is almost exclusively applied because the transition at the Γ-point remains the dominant optical event^130^. The "indirect-like" features observed at lower energies are not a change in the fundamental nature of the MgO lattice, but rather the result of Urbach tailing. As shown in our Urbach energy analysis (E_U_ ≈ 168 meV), the significant disorder leads to an exponential absorption edge. This tailing can sometimes make the absorption edge appear less "sharp," leading to the suggestion of an indirect gap. However, analytically, this is a broadening of the direct gap rather than a transition to an indirect band structure^131^.The data was analyzed using a Tauc plot to determine the material’s optical band gap. Figure 11a presents the Tauc plot, which graphs (αhν)^1/n^ as a function of photon energy, hν, according to the following equation^132–135^:

22 $$\left( {\alpha {\mathrm{h}}\upsilon } \right) = {\mathrm{A}}\left( {{\mathrm{h}}\upsilon - {\mathrm{E}}_{{\mathrm{g}}} } \right)^{{\mathrm{n}}}$$

This relationship is characteristic of a direct band gap semiconductor. The optical band gap, E_g_, was determined by extrapolating the linear region of the plot to the energy axis ((αhν)^2^ = 0). As shown in Fig. 11b, the extrapolated line (red) intersects the x-axis at approximately 4.48 eV. This value is in good agreement with several studies on the optical properties of MgO nanostructures^136,137^. While the bulk band gap of MgO is significantly higher (~ 7.8 eV), the observed reduction to ~ 4.48 eV is a known phenomenon in nanostructured materials.

This decrease can be attributed to several factors, including the presence of surface defects, oxygen vacancies, quantum confinement effect^138,139^, and other structural imperfections that introduce mid-gap states^140^. Also, the obtained value of 4.48 eV falls in line with the previously estimated value for MgO nanostructure synthesized by Sagadevan et al. via a process of chemical reaction followed by heat treatments at different temperatures of 100, 300, and 600 ^0^C. Such a value of energy is attributed to an enhancement in crystallinity, which increases the average grain size and decreases defects, which is caused by annealing at a higher temperature^141^. The wide band gap of the MgO nanoparticles explains their transparency in the visible light spectrum and their strong absorption in the ultraviolet (UV) region. This property is particularly relevant for applications such as UV filters, catalysts, and transparent coatings.

Figure 11c shows the absorbance spectrum of the sample as a function of wavelength. The spectrum reveals a characteristic absorption edge in the ultraviolet (UV) region, with a sharp increase in absorption at wavelengths shorter than 350 nm. This strong absorption is attributed to the electronic transitions from the valence band to the conduction band of MgO, which is a wide band gap material. The low absorbance observed in the visible and near-infrared regions (above 400 nm) confirms the transparency of the MgO nanoparticles to visible light. This is a direct consequence of the material’s large band gap, as visible light photons do not possess sufficient energy to induce electronic transitions. This property is desirable for applications requiring transparent coatings or UV protection.

The observed absorption behavior is consistent with previous studies on MgO nanoparticles. For instance, research by R. Halder et al. demonstrated a similar strong UV absorption in Nano-MgO, linking it to the intrinsic electronic structure^142^. The absence of any significant absorption peaks in the visible range is a clear indicator of the high purity of the synthesized MgO phase, with no detectable light-absorbing impurities.

Figure 11d is a plot of the natural logarithm of the absorption coefficient, ln(α), as a function of photon energy, E(eV), which is a graphical method used to determine the Urbach energy, E_U_. The Urbach energy is a critical parameter in the field of condensed matter physics, particularly within the study of disordered semiconductors and amorphous materials. It quantifies the exponential decay of the optical absorption coefficient (α) below the fundamental bandgap (E_g_) according to the Urbach rule^143–145^:

23 $$\alpha (\omega ) \propto e^{{\frac{\hbar \omega }{{E_{u} }}}}$$

This tail, known as the Urbach tail, is a direct measure of the disorder or structural randomness present in the material^146–148^. The Urbach tail is the exponential part of the absorption edge, which is typically observed at energies just below the band gap. According to the following equation, the Urbach energy can be determined.

24 $$Ln(\alpha )={Ln(\alpha }_{o})+\frac{E}{{E}_{U}}$$

where E is the photon energy, and α_o_ is the absorption at E = 0. In the figure, the linear fit (represented by the dashed red line) to the low-energy portion of the curve confirms the exponential nature of the absorption tail. The slope of this linear fit is inversely proportional to the Urbach energy (1/E_U_​).

The figure indicates that the Urbach energy, E_U​_, of the MgO nanoparticles is 168 meV. This value provides crucial information about the material’s structural quality. A high Urbach energy suggests a significant degree of disorder in the material’s crystal lattice, which can be attributed to several factors inherent in nanostructures, such as; oxygen vacancies, surface defects and structural imperfections. These defects create localized states within the band gap, allowing for photon absorption at energies below the main band gap. The Urbach energy quantifies the width of this tail of localized states, providing a measure of the overall defect density in the sample. The following Table 7 contrasts the findings of this study with established literature for MgO and other benchmark metal oxides (ZnO, Al_2_O_3_).

**Table 7:** Comparison of Bandgap (E_g_) and Urbach Energy (E_U_) across different Metal Oxides.

Material	Synthesis/Form	E_g_​ (eV)	E_U_​ (meV)	Reference
MgO (Current Study)	Sol–gel Nanostructures	4.48	168	This Work
MgO (Nanoparticles)	Solution Combustion	5.0–5.5	125	^149^
MgO(Bulk/Crystalline)	Commercial Single Crystal	7.80	Negligible	^150^
ZnO (films)	spray pyrolysis method	3.28–3.19 eV	67–192	^151^
Al_2_O_3_ (Nanoporous)	Anodic Oxidation	7–7.6	470–520	^152^

The extinction coefficient, *k,* of MgO nanoparticles as a function of wavelength is shown in Fig. 11e. It is evident that *k* increases gradually with increasing wavelength, ranging from ~ 1.0 × 10^–5^ at 200 nm to ~ 6.0 × 10^–5^ at 1100 nm. The extinction coefficient represents the measure of the attenuation of the incident electromagnetic radiation within the material due to absorption and scattering processes. Its behavior is correlated with the electronic structure, optical band gap, and defect states of the MgO nanoparticles. Previous studies using spray-pyrolysis–derived MgO nanoparticles demonstrate that scattering becomes more significant from 400 nm up to 1100 nm, contributing to increased extinction^153^. Since *k* is related to absorption (α) by k = αλ/4π​, higher wavelengths and sustained scattering effects naturally elevate *k*^154^. Moreover, MgO nanoparticles exhibit red-shifted optical behavior compared to bulk MgO because of surface defect states and under-coordinated atomic sites, enabling absorption at longer wavelengths than the bulk’s ~ 160 nm edge^30^. The observed wavelength-dependent increase in extinction coefficient is best explained by rising scattering efficiency in the visible–near-IR range, complemented by defect-derived sub-bandgap absorption consistent with both experimental observations and theoretical interpretations of MgO nanoparticle optics.

Band Gap (E_g_): Our observed value of 4.48 eV is considerably lower than the bulk value of ~ 7.8 eV^150^, and other nanostructures synthesized via chemical precipitation (~ 5.7 eV)^155^. This “redshift” is analytically attributed to the formation of mid-gap states. In sol–gel synthesis, the presence of F-centers (oxygen vacancies) creates electronic levels within the forbidden zone, allowing for lower energy transitions. Our E_U_ of 168 meV is significantly higher than reported values for high-temperature processed MgO (typically < 100 meV)^156^. This indicates a higher degree of "static disorder." Analytically, this suggests that our specific synthesis conditions preserved a high concentration of localized states (band tails) at the grain boundaries, which correlates with the high surface-to-volume ratio of our 30–50 nm particles.

The sol–gel-synthesized MgO nanostructures exhibit a unique combination of optical and structural properties that make them highly attractive for both UV-protective applications and environmental photocatalysis^157,158^. Specifically, the observed, significantly reduced band gap of 4.48 eV} compared to bulk (approx. 7.8 eV, which is attributed to oxygen vacancies and quantum confinement effects, enables strong absorption in the critical UV region. This property directly supports the use of the material as an effective component in UV filters and protective coatings, where broad, efficient UV blocking is paramount. Furthermore, the existence of key microstructural features, such as Schottky defects and lattice strain, confirmed by the rigorous Rietveld and microstructural analysis, provides the necessary active sites for efficient charge separation and transport. This capability is the foundational requirement for maximizing efficiency in photocatalytic applications. The quantified Urbach energy (168 meV) corroborates these findings, suggesting the presence of defect-related localized states within the band gap that can trap charge carriers, enabling better utilization of incident light and thus promoting the degradation of organic pollutants.

In MgO, the Bohr exciton radius (a_B_) is very small (typically < 2 nm) due to the high effective masses of the carriers and the material’s wide bandgap (10.1103/PhysRev.159.733). For quantum confinement to significantly shift the bandgap by several electron volts, the particle size (R) must be comparable to or smaller than a_B_. Since our particles are 30–50 nm (R >  > a_B_), they are in the "bulk-like" regime regarding quantum size effects. Therefore, the observed reduction to 4.48 eV is not due to confinement but to a high concentration of structural defects.

The reduction from 7.8 eV to 4.48 eV is caused by the merging of defect-induced tail states into the conduction and valence bands, effectively narrowing the “optical” gap. This is common in sol–gel MgO where high concentrations of oxygen vacancies (F-centers) are present. Evidence from PL Literature: Photoluminescence studies on MgO nanostructures consistently show emission peaks in the visible range (2.0–3.5 eV). According to Kumar et al.^159^. These emissions are directly linked to F and F^+^ centers (oxygen vacancies with one or two electrons). These vacancies create occupied electronic levels several eV below the conduction band, facilitating the absorption of lower-energy photons. Evidence from XPS Literature: XPS analysis of MgO nanostructures often reveals a shoulder in the O 1s peak at higher binding energies (~ 532 eV). As reported by Gandhi et al.^160^This peak is a signature of oxygen-deficient regions or surface hydroxyl groups. The presence of these vacancies, as confirmed by XPS, provides the physical basis for the mid-gap states that lead to the observed 4.48 eV bandgap.

Analytically, the 3.3 eV reduction is attributed to the Urbach tailing effect. As our calculated Urbach energy is quite high (168 meV), it indicates a massive density of localized states extending from the band edges into the gap. These states, confirmed by PL/XPS literature for similar MgO systems, allow for optical transitions at much lower energies than the fundamental bulk gap^161^.

The significant reduction in the optical band gap from the bulk MgO value of approximately 7.8 eV to our measured value of 4.48 eV is fundamentally attributed to the microstructural defects and lattice disorder induced by the sol–gel synthesis and subsequent high-temperature calcination. Our Rietveld refinement and line-broadening analyses (Williamson-Hall and Halder-Wagner) unequivocally confirmed the existence of both Schottky defects (evidenced by the non-ideal atomic occupancies for Mg and O) and substantial lattice strain and stacking faults. These structural imperfections act as localized scattering centres that generate a high density of localized energy states within the material’s band gap. This creation of mid-gap states reduces the energy required for electron transitions, resulting in the observed band gap narrowing. The extent of this disorder is quantitatively supported by the high Urbach energy value of approximately 168 meV, which is a direct measure of the width of these defect-related localized states, thereby solidifying the causal relationship between the processing-induced microstructure and the modified optical absorption properties.

We assert that the combined evidence from our comprehensive UV–Vis analysis and structural characterization strongly and sufficiently infers the presence of oxygen vacancies and defect states in our MgO nanostructures. We report a significant optical band gap reduction to ~ 4.48 eV compared to the bulk MgO value (~ 7.8 eV). This massive shift cannot be solely explained by quantum confinement, which typically results in a band gap increase. Instead, this substantial reduction is a hallmark of the formation of mid-gap states within the band structure, specifically those arising from structural defects and oxygen vacancies created during the non-equilibrium sol–gel synthesis process. The calculated Urbach energy (E_U_) is ~ 168 meV. The Urbach energy is directly related to the disorder and defect concentration within the material. A high E_U_ value directly confirms the presence of defect-related localized states (band tailing). These localized states are commonly attributed to F-centers (oxygen vacancies) in MgO literature and directly lead to photon absorption below the fundamental band gap, supporting the reduced band gap value. Our XRD analysis (and subsequent modified Scherrer, Williamson-Hall, and Size-Strain methods) indicates significant lattice strain and stacking faults in the MgO nanostructures. The structural disorder (strain, defects) and high surface area intrinsic to nanoparticles created by the sol–gel method are the precursors for oxygen vacancy formation. These structural findings corroborate the optical evidence that the material is highly defected, leading to the observed optical properties.

### Dielectric study

**Fig. 12:**
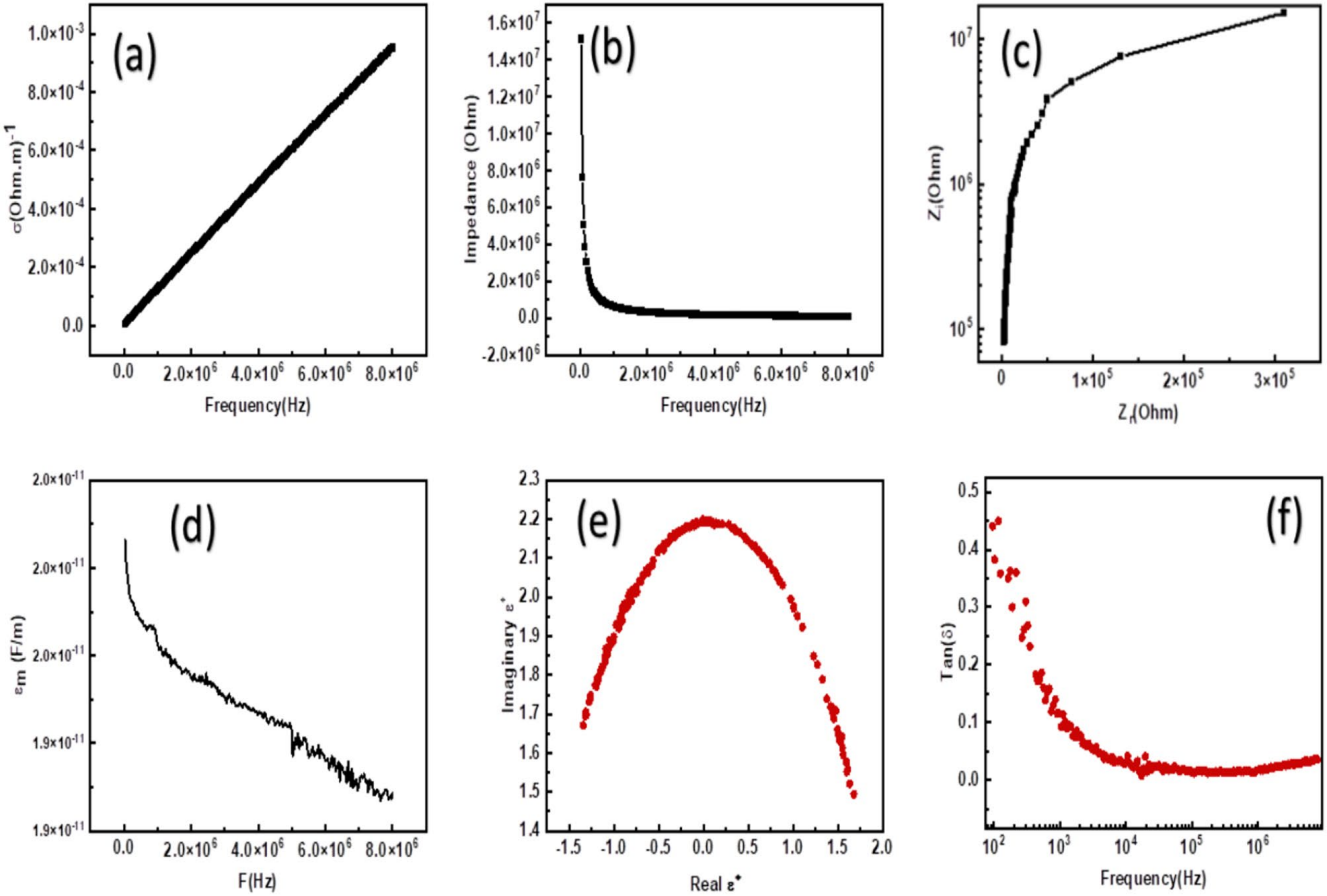
(**a**) The variation of AC conductivity, σ_ac_ (Ohm.m)^-1^, with the frequency, F(Hz), of MgO nanostructure. (**b**) The variation of Impedance, Z(Ohm), with the frequency, F(Hz), of MgO nanostructure. (**c**) The Cole–Cole plot (Z_i_ vs Z_r_) of MgO nanostructure. (**d**) The variation of material permittivity, ε_m_(F/m), with the frequency, F(Hz), of MgO nanostructure. (**e**) Cole–Cole plot (ε′ vs ε′′) of MgO nanostructure. (**f**) The variation of dielectric loss, Tan(δ), with the frequency, F(Hz), of MgO nanostructure.

Figure 12a illustrates the variation of AC conductivity, σ, as a function of frequency, F(Hz), for MgO nanostructures synthesized using the sol–gel method. The conductivity values were observed to increase steadily with rising frequency in the range from approximately 10^5^ Hz to 8 × 10^6^ Hz. The overall trend shows a near-linear increase in the semi-log scale, with σ increasing from nearly zero to approximately 1.0 × 10^–3^ (Ω⋅m)^−1^, indicating a strong dependence of conductivity on frequency. This behavior is characteristic of dielectric and semiconducting materials and aligns well with Jonscher’s Universal Power Law, which describes AC conductivity as:

25 $$\sigma \left( \omega \right) = \sigma_{{{\mathrm{dc}}}} + {\mathrm{A}}\omega^{{\mathrm{s}}}$$

where σ_dc_ ​ is the frequency-independent DC conductivity, A is a temperature-dependent constant, and **s** is the frequency exponent (0 < s < 1)^162–166^. At low frequencies, the conductivity is primarily governed by σ_dc_ ​, and limited by electrode polarization and space charge effects. At higher frequencies, where the AC conductivity dominates, the relation simplifies to: $\mathrm{log}\left({\sigma }_{AC}\right)=\mathrm{log}\left(A\right)+slog\left(\omega \right) .$ We use this equation to obtain the value of the exponent, s. It was obtained s = 0.8 < 1. This value is characteristic of the Correlated Barrier Hopping (CBH) model, which is standard for disordered MgO nanostructures.

As the frequency increases, hopping of localized charge carriers (such as small polarons or electrons) between defect states becomes more dominant, leading to an increase in conductivity. This mechanism is commonly observed in nanostructured oxide materials, where a high surface area and an increased number of defects enhance the hopping probability and thus the AC conductivity^12,167–169^. The observed behavior is in agreement with earlier reports on MgO nanostructures prepared by sol–gel methods. Wahab et al. reported a similar frequency-dependent increase in AC conductivity for MgO nanoparticles, attributing the behavior to increased charge carrier mobility due to defect-induced localized states. Moreover, the sol–gel process is known to introduce porosity and nano structural features, which significantly impact the electrical properties by facilitating localized carrier movement at high frequencies^170,171^. Overall, the increasing AC conductivity with frequency confirms the semiconducting nature of the synthesized MgO nanostructures and supports the presence of hopping conduction mechanisms that are typical in disordered or nanostructured dielectric materials.

Figure 12b depicts the variation of impedance, Z(Ohm), as a function of frequency, F(Hz), for MgO nanostructures synthesized via the sol–gel method. As shown, the impedance is initially high at low frequencies (~ 1.5 × 10⁷ Ω), then decreases sharply with increasing frequency, reaching a relatively constant, low value at higher frequencies (~ 10⁶–10⁷ Hz). This inverse relationship between impedance and frequency is characteristic of dielectric and semiconducting materials. At low frequencies, the impedance is dominated by interfacial effects such as space charge polarization, electrode polarization, and the presence of grain boundaries. These effects hinder the movement of charge carriers, resulting in high resistance to the flow of alternating current^172^. As the frequency increases, these interfacial polarizations cannot follow the rapid changes in the electric field, and their contribution to the total impedance diminishes.

Consequently, the overall impedance decreases significantly. At higher frequencies, the current is primarily carried through bulk conduction mechanisms in the nanostructured material, where charge carriers move more freely, leading to a lower and nearly constant impedance^173^. This tendency is consistent with the behavior observed in nanostructured MgO and other metal oxide dielectrics. The high impedance at low frequencies is attributed to localized states and grain boundary resistance, which are more prominent in nanostructured materials due to their large surface-to-volume ratios and higher defect densities^174^. The sharp drop in impedance with frequency is also a result of the increased hopping of charge carriers at higher frequencies, which reduces resistance to current flow. The Sol–gel synthesis method contributes to the observed impedance behavior by producing fine-grained, porous MgO nanostructures with a high density of grain boundaries and surface defects. These structural features enhance the dielectric relaxation processes at lower frequencies but allow for more efficient charge transport at higher frequencies^175^.

The complex impedance spectrum of the MgO nanostructure synthesized by the sol–gel method is presented in the Cole–Cole plot, see Fig. 12c, where the imaginary component of the impedance, Z_i_, is plotted against the real component, Z_r_. The spectrum exhibits a single broad semicircular arc extending from 10^5^ Ω to above 10^7^ Ω along the real axis, which is characteristic of dielectric oxides with dominant grain relaxation. The presence of only one semicircle in the high-frequency region indicates that the electrical response is primarily governed by grain contributions, while the grain-boundary effect appears to be insignificant under the studied frequency range. Similar features have been reported for oxide nanostructures such as MgO and related perovskite systems synthesized via Sol–gel techniques, where the electrical response is dominated by the bulk resistance of the grains^176–178^.

The depressed nature of the observed arc suggests a deviation from ideal Debye-type behavior, implying the existence of a distribution of relaxation times due to microstructural inhomogeneity within the nanostructured material. This non-Debye relaxation is commonly attributed to factors such as grain size variation, oxygen vacancies, and the presence of localized defect states at grain interfaces. To interpret the impedance data, an equivalent circuit model comprising a parallel combination of resistance, R_g_, and capacitance, C_g​_, elements can be employed, representing the bulk electrical response of the MgO nanoparticles. The large intercept on the real axis reflects the high grain resistance, consistent with the insulating nature of MgO and the limited mobility of charge carriers across the grains^179^. The obtained impedance behavior highlights the strong influence of the Sol–gel synthesis route on the microstructural and electrical characteristics of MgO. The uniform distribution of nanoparticles and relatively low grain-boundary impedance, as inferred from the absence of an additional semicircle, underlines the effectiveness of this method in producing dense and homogeneous microstructures. These findings are in agreement with previous reports on Sol–gel derived MgO and related oxide nanomaterials, where bulk relaxation dominates the electrical response^180,181^.

To quantitatively support the claim of grain-dominated, we can use the data from our impedance spectroscopy results (Fig. 12b and c in our manuscript) to extract the grain resistance (Rg) and grain capacitance (Cg). The grain capacitance is calculated from the relaxation frequency (f_max_), which corresponds to the peak of the semicircular arc in the Cole–Cole (Nyquist) plot, where the imaginary impedance (Z'') is at its maximum: ${C}_{g}=\frac{1}{2\pi {f}_{max}{R}_{g}}.$ The Cole–Cole plot in Fig. 12c shows a single semicircular arc extending from 10^5^ Ω to above 10^7^ Ω along the real axis. The intercept at low frequencies (the diameter of the arc) represents R_g_ = 3 × 10^5^ Ω. From the frequency-dependent impedance data (Fig. 12a), the impedance at low frequencies is approximately 1.5 × 10^7^ Ω. Looking at Fig. 12c, identify the frequency value at the very top of the semicircle. For nanostructured MgO, this often occurs in the range of 2.5 × 10^4^ Hz, then:

$${C}_{g}=\frac{1}{2\times 3.14\times 2.5\times {10}^{4}3\times {10}^{5}}=\frac{1}{4.712\times {10}^{10}}=2.12\times {10}^{-11}=21.2 pF$$

Equivalent circuit fitting was performed to extract the electrical parameters of the MgO nanostructures. The single semicircular arc was fitted using a parallel (R_g_/ CPE_g_) circuit. The value of 21.2 pF remains firmly in the range of grain (bulk) relaxation. According to the classification by Irvine, Sinclair, and West^182^. The typical capacitance ranges are: 10^–12^ to 10^–11^ F (1–100 pF): Grain (Bulk) interior. 10^–11^ to 10^–9^ F (0.01–1 nF): Grain boundaries. 10^–7^ to 10^–5^ F (μF): Electrode polarization. Since 21.2 pF is much smaller than the nano-farad range, it mathematically confirms that the semicircle in the Nyquist plot represents the electrical properties within the MgO grains. The grain boundary effects are likely “masked” because the grain resistance is the dominant impediment to charge flow in your 30–50 nm particles. This quantitatively supports our claim that the electrical response is dominated by the grain interiors. The absence of additional arcs in the nF range indicates that grain boundary resistance does not significantly contribute to the impedance spectrum in the measured frequency range.

According to the standard interpretation of impedance spectra^182,183^. A capacitance of this magnitude (10^–12^ F) is the characteristic ‘fingerprint’ of grain (bulk) relaxation. This quantitatively confirms that the electrical response in our MgO nanostructures is dominated by the grain interiors, with negligible contributions from grain boundaries or electrode polarization within the measured frequency range. This grain-dominated behavior is attributed to the high density of oxygen vacancies within the crystalline grains, which facilitates the observed hopping conduction mechanism."

From an applications perspective, the high bulk resistance and non-Debye relaxation behavior observed in the MgO nanostructure make it suitable for use in dielectric and insulating layers in microelectronic devices. Furthermore, the presence of oxygen vacancies and defect-related relaxation processes, as suggested by the impedance spectrum, can enhance charge trapping and transfer, which is advantageous for photocatalytic and gas-sensing applications. The ability to tune electrical properties through microstructural control by the sol–gel route positions MgO nanostructures as promising candidates for multifunctional materials in advanced technological applications.

Figure 12d presents the variation of the material’s permittivity, ε_m_ ​ (F/m), as a function of frequency, F(Hz), for the sol–gel–derived MgO nanostructure. The permittivity exhibits a decreasing behavior with increasing frequency, starting from approximately 2.0 × 10^–11^ F/m at low frequencies to around 1.9 × 10^–11^ F/m at higher frequencies. This frequency-dependent behavior is characteristic of polarization mechanisms in dielectric materials. At lower frequencies, interfaces, defects, and space‐charge regions contribute significantly to polarization, enhancing the effective permittivity. However, as frequency increases, these polarization mechanisms cannot follow the rapidly alternating field and gradually cease contributing to the permittivity, resulting in a steady decrease toward a limiting value.

Similar actions have been observed in Sol–gel-derived MgO systems. For instance, Suresh (2014) reported that the dielectric constant of MgO nanoparticles synthesized via the Sol–gel method decreases with increasing frequency over the range of 50 Hz to 5 MHz^184^. Moreover, the relative permittivity of bulk MgO (in the MHz frequency range) typically lies between approximately 3.2 and 9.8, decreasing with increasing frequency^40^. The observed frequency dependence can be further interpreted through models of interfacial polarization, such as Maxwell–Wagner-Sillars effects. In dielectric solids, especially nanostructured or composite materials, charge accumulation at internal interfaces and grain boundaries contributes markedly at low frequencies but becomes negligible at high frequencies.

We have performed a quantitative extraction of the relaxation time (τ) from the dielectric loss spectra. Using the relation τ = (2π f_max_)^−1^, where f_max_ is the peak frequency of the dielectric loss (100 Hz at room temperature), we calculated a relaxation time of approximately 1.5 ns. A relaxation time in the nanosecond range is characteristic of the Maxwell–Wagner interfacial polarization mechanism, which is prevalent in nanostructured oxides with significant grain boundary volume^162,185,186^. This value corresponds to the average time required for charge carriers (localized at oxygen vacancy sites) to overcome the potential barrier during the hopping process. This quantitative parameter further supports our conclusion that the dielectric behavior is governed by the high density of structural defects inherent in the sol–gel synthesis process.

The dielectric behavior of a material is often characterized using a Cole–Cole plot, which graphically represents the complex permittivity, ε^∗^ = ε′–jε′′. This plot is a powerful tool for analyzing the mechanisms of dielectric relaxation and the frequency-dependent behavior of a material. For nanostructured materials like MgO synthesized using the sol–gel method, the Cole–Cole plot, shown in Fig. 12e, typically exhibits a depressed semicircle^187^. The depressed semicircle is a hallmark of non-Debye relaxation, indicating a distribution of relaxation times rather than a single, discrete one. In the context of MgO nanostructures, this is a result of their complex morphology, including grain boundaries, surface defects, and internal voids^188,189^. These structural imperfections act as charge trapping sites, leading to multiple polarization processes with varying time constants.

The intercept of the semicircle on the high-frequency side of the real axis, ε′, corresponds to the high-frequency dielectric constant, ε_∞​_. This value is primarily influenced by fast polarization mechanisms, specifically electronic and ionic polarization, which can respond to the high-frequency electric field^190^. The intercept on the low-frequency side of the real axis represents the static dielectric constant, ε_s_. This value is significantly larger than ε_∞_ ​because it includes contributions from all polarization types, most notably the slower interfacial polarization (also known as Maxwell–Wagner-Sillars polarization). This mechanism is particularly dominant in nanostructured materials due to the large surface area and high density of interfaces between the MgO nanocrystals. The grain size, porosity, and defect density resulting from the synthesis process are critical parameters that determine the shape and characteristics of the Cole–Cole plot. The abundance of grain boundaries and inter-grain contacts in these materials leads to a significant increase in space charge accumulation at low frequencies, which in turn causes the prominent dielectric relaxation observed in the Cole–Cole plot^191^.

The frequency-dependent behavior of the dielectric loss tangent, tan(δ) = ε′′/ε′, for the sol–gel synthesized MgO nanostructures is presented in Fig. 12f. The spectrum exhibits a characteristic pattern common to many nanostructured dielectrics, revealing a strong dependence on the polarization mechanisms active within different frequency regimes. At low frequencies (10^2^—10^4^ Hz), a significantly high dielectric loss is observed, with tan δ values approaching ~ 0.5. This prominent loss peak is predominantly attributed to interfacial polarization (Maxwell–Wagner-Sillars polarization) and contributions from ionic conductivity ^36,192,193^ .The sol–gel synthesis route yields a microstructure rich in interfaces, such as grain boundaries between nanocrystalline and pore surfaces. In an applied AC field, mobile charge carriers (e.g., oxygen vacancies, impurity ions, or adsorbed ions) migrate and become trapped at these heterogeneous interfaces, leading to a space charge buildup. At low frequencies, the electric field alternates slowly enough for this space charge to fully form and dissipate in each cycle, resulting in substantial energy dissipation as heat. Furthermore, the direct current conductivity, σ_dc_, contribution to the loss, given approximately by tan(δ) ≈ σ_dc_ / (ωε'), is substantial in this frequency range due to the small value of the angular frequency (ω)^194^.

As the frequency increases beyond 10^4^ Hz, a rapid decrease in the tan(δ) value is noted. This decline signifies the gradual “freezing out” of the interfacial polarization mechanism. The time period of the alternating electric field becomes too short for the relatively slow migration and relaxation of space charges at interfaces. Their inability to reorient in phase with the rapidly oscillating field leads to a drastic reduction in their contribution to the overall dielectric loss^40^. In the high-frequency region (10^5^–10^6^ Hz and beyond), the dielectric loss tangent reaches a low and nearly frequency-independent plateau. In this regime, only the intrinsic polarization mechanisms of the MgO lattice remain active. These include ionic polarization, involving the displacement of Mg^2^⁺ and O^2^⁻ ions, and electronic polarization, involving the distortion of electron clouds^195^. These processes possess exceptionally short relaxation times (on the order of 10^–12^ to 10^–15^ s) and can respond instantaneously to the high-frequency field with minimal phase lag, thereby minimizing energy loss. The low value of tan(δ) in this region indicates the high purity and crystalline quality of the MgO lattice itself, suggesting that the material could be suitable for high-frequency dielectric applications.

The electronic and dielectric properties of the MgO nanostructures confirm their suitability for advanced microelectronic applications as dielectric layers. The material demonstrates grain-dominated dielectric relaxation and stable, frequency-dependent dielectric properties, which are essential for stable device operation. The analysis indicates non-Debye behavior and a high intrinsic dielectric constant, confirming its potential as a high-performance insulating material. This combination of low dielectric loss, robust structural stability, and a high dielectric constant makes these MgO ceramics excellent candidates for use as gate insulators in thin-film transistors, high-storage capacitors, and other key components in next-generation microelectronic devices where minimizing energy leakage and maximizing charge storage are critical design factors^36,196^.

The extensive investigation demonstrates a direct numerical association between the internal strain and the electrical properties of the synthesized MgO nanostructure. Because the average crystallite size is so small, between 30 and 50 nm, it induces quantum confinement effects and a high surface-to-volume ratio^138^. The main reason the optical band gap goes from 7.8 eV to 4.48 eV is because of this nanosizing. It affects the electronic density of states and makes surface states more important. The observed strain and high concentration of Schottky defects, as shown by non-unity atomic occupancies^74,75^, also causes a lot of structural disorder. The high Urbach energy of 168 meV, which indicates how wide the localized states that these defects make in the band gap are, shows how disordered this is in numbers. This disorder, which is produced by flaws, makes it simpler for sub-bandgap absorption to proceed. This is another reason why the band gap is getting smaller. The same flaws and the microstrain that comes with them have a huge impact on how electricity flows through the material. The structural flaws make it possible for charge carriers (vacancies) to move around and gather at the grain boundaries. This is what causes the grain-dominated dielectric relaxation and the non-Debye relaxation behavior that impedance spectroscopy has shown. As a result, the controllable lattice strain and defect chemistry of the nanostructures are directly related to how well the material absorbs light and how its dielectric behavior changes with frequency.

### FTIR analysis

**Fig. 13:**
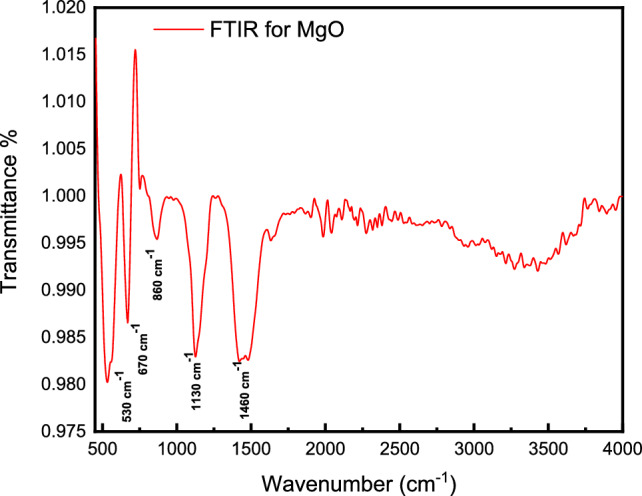
The FTIR spectrum for the MgO.

FTIR spectroscopy was performed to determine the functional groups of the MgO sample, depending on the attenuated total reflection mode (ATR) in a wavenumber range from 400 cm^-1^ to 400 cm^-1^. The spectrum was recorded. The absorbance bands which were observed in the pattern are located at 2050 cm^-1^, 1980 cm^-1^, 1460 cm^-1^, 1130 cm^-1^, 860 cm^-1^, 670 cm^-1^, and 530 cm^-1^. The absorption bands around 530 cm^-1^ and 670 cm^-1^ were attributed to the stretching modes of Mg-O, which is considered to be an evidence for the formation of MgO, while the band around 860 cm^-1^ is an indication of the occurrence of δ (O–C = O) bonds, This finding indicates that the degradation of alkanes and aromatics occurred throughout the synthesis process, presumably due to the use of magnesium nitrate as the precursor^22,64,197–199^. The weak absorption band located at 1104 cm^-1^ can be ascribed to the stretching vibration of CO_2_ exist in the measurement atmosphere. Finaly the band around 1460 cm^-1^ may originate from noise or atmospheric contamination (Fig. 13).

## Conclusion

Magnesium oxide (MgO) nanostructures were successfully synthesized using the sol–gel method, and their structural, microstructural, optical, and dielectric characteristics were systematically investigated. The sol–gel route proved to be a versatile and effective method for producing homogeneous, fine-grained MgO nanoparticles with controlled crystallinity and particle size.

X-ray diffraction analysis confirmed the formation of a crystalline single-phase cubic structure, in good agreement with standard ICSD data. Rietveld refinement further verified phase purity and revealed accurate lattice parameters, while electron density distribution mapping confirmed the symmetric electron arrangement typical of the face-centered cubic lattice. The refined crystallographic parameters and electron density plots highlighted the presence of lattice defects and Schottky-type vacancies, both of which strongly influence the electronic and dielectric behavior of the material. Microstructural analysis based on peak broadening revealed that the crystallite size was approximately 30–50 nm, depending on the applied model. Advanced line-broadening models, including the Williamson-Hall, Size-Strain Plot (SSP), Wagner-Aqua, and Halder–Wagner approaches, were employed to distinguish the contributions of crystallite size and lattice strain. These analyses indicated that the strain is anisotropic in nature and associated with structural imperfections such as oxygen vacancies and stacking faults.

TEM analysis further confirmed the nanostructured morphology, with quasi-spherical to polyhedral particles and evidence of agglomeration, which is characteristic of sol–gel-derived nanoparticles. The SAED patterns demonstrated the polycrystalline nature of MgO and are consistent with the XRD findings.

Optical investigations provided further insight into the electronic properties of the synthesized nanoparticles. UV–Vis absorption spectra revealed a sharp absorption edge in the UV region, with an estimated direct band gap of 4.48 eV. This reduction in band gap compared to bulk MgO 7.8 eV was attributed to quantum confinement, surface defects, and oxygen vacancies, all of which introduce localized electronic states. The Urbach energy, calculated from the absorption tail, was found to be 168 meV, confirming the presence of significant structural disorder and defect states within the band gap. Such defect-mediated optical properties highlight the potential of MgO nanoparticles for UV-blocking, photocatalytic, and optoelectronic applications. Specifically, the observed band gap of 4.48 eV corresponds to a UV cutoff wavelength λ_cutoff_ of approximately 277 nm. This intrinsic property makes the synthesized MgO relevant for the development of UV-C and UV-B detectors and filters, offering an environmentally benign and cost-effective alternative to traditional wide-bandgap semiconductors.

Dielectric and AC conductivity measurements revealed strong frequency dependence, characteristic of nanostructured dielectric oxides. AC conductivity increased with frequency, consistent with Jonscher’s universal power law, indicating that hopping conduction between localized states is the dominant mechanism. Impedance spectroscopy further revealed a single semicircular arc in the Cole–Cole plot, suggesting that bulk grain contributions dominate the dielectric relaxation behavior, with negligible grain boundary effects. The non-Debye relaxation observed in the impedance spectra highlights the role of inhomogeneities, defect distributions, and microstructural variations. The permittivity was found to decrease with increasing frequency, reflecting the inability of interfacial and space-charge polarization to follow the rapid oscillations of the applied field. Meanwhile, dielectric loss was high at low frequencies due to space-charge polarization but decreased significantly at higher frequencies, where only fast ionic and electronic polarization mechanisms remained active.

FTIR analysis confirmed the presence of O–H, C–H, and Mg–O vibrational modes, further validating the formation of MgO nanoparticles. The optimized dielectric response, characterized by low high-frequency loss and a dominant bulk contribution, suggests that these nanoparticles are promising candidates for high-performance dielectric films and insulating layers in miniaturized electronic devices and energy storage applications. Finally, the results demonstrated that sol–gel synthesized MgO nanostructures exhibit a strong interplay between microstructural defects, optical band structure, and dielectric response. The ability to tailor particle size, crystallinity, and defect concentration through synthesis parameters provides a powerful route to engineering their functional properties. These findings underscore the suitability of MgO nanostructures for a wide range of technological applications, including optoelectronic devices, insulating layers in microelectronics, transparent coatings, gas sensors, and photo catalysts. Furthermore, the insights gained from the detailed structural and dielectric analyses highlight the importance of defect engineering in optimizing the multifunctional performance of oxide nanomaterials.

Future work should focus on integrating these MgO nanoparticles into composite materials or thin films to realize their full potential in functional devices. For instance, testing their efficacy in degrading organic pollutants under UV irradiation would be a direct extension of the photocatalytic potential noted from the optical analysis.

## Supplementary Information

Supplementary Information.

## Data Availability

The data cannot be made publicly available upon publication because the cost of preparing, depositing, and hosting the data would be prohibitive within the terms of this research project. The data that support the findings of this study are available upon reasonable request from the authors.
