# Supplementary material for: Sol–gel synthesis and comprehensive characterization of MgO nanostructures: structural, optical, and dielectric insights
Source: Sci Rep. 2026 Apr 13;16:12215. doi: 10.1038/s41598-026-44397-5 (PMC13077086; doi:10.1038/s41598-026-44397-5)
Supplement: Supplementary file 1 — Supplementary Information. [file 41598_2026_44397_MOESM1_ESM.docx]

**Supplementary Information**

**Sol-gel synthesis and comprehensive characterization of MgO nanostructures: structural, optical, and dielectric insights**

Naglaa AbdelAll^1^, Asmae Mimouni^2^, Abdalrahman M. Rayan^3^, Ghada A. Khouqeer^2^, Mohamed Asran Hassan^3^ and Mahrous R. Ahmed*^3^

^1^Physics Department, Faculty of Science, Assiut University, Assiut 71516, Egypt

^2^ Physics Department, Faculty of Science, Imam Mohammad Ibn Saud Islamic University (IMSIU), Riyadh 11564, Saudi Arabia

^3^Physics Department, Faculty of Science, Sohag University, Sohag 82524, Egypt

***Correspondence Authors:**

Mahrous R. Ahmed: mahrous.r.ahmed@science.sohag.edu.eg

We here briefly discuss theoretical derivations of the Rietveld refinement done for the studied sample; the equations are included here to enhance the readability of the article itself.
The Rietveld refinement utilizes the Pseudo-Voigt function, which serves as the line broadening β(2θ) function. This function primarily illustrates a peak that amalgamates two profiles: Lorentzian and Gaussian. The subsequent Eq.(S1) delineates the Pseudo-Voigt function as follows

PV(X) = η L(X) + (1-η) G(X) (S1)

Where η represents the enhancement percentage of the function, G(X) denotes the Gaussian contribution, and L(X) signifies the Lorentzian contribution to broadening.

For the R-factors, also known as Residual factors, sometimes referred to as reliability factors, are mathematical indicators utilized in Rietveld refinement to assess the degree of fit between the estimated pattern derived from the structural model and the observed diffraction pattern. These factors are utilized to assess the degree to which the enhanced model aligns with the experimental results. In Rietveld refinement, the predominant R-factors utilized are: Rₚ (Profile R-factor) quantifies the concordance between the observed and computed diffraction profiles. This can be expressed in Eq.(S2) In Rietveld refinement, the predominant R-factors utilized are: Rₚ (Profile R-factor) quantifies the concordance between the observed and computed diffraction profiles. This can be expressed in Eq. (S2).

*R_p_* =100x $\frac{\sum Y_{Obs}- Y_{Calc}}{\sum Y_{Obs}}$ (S2),

Y_Obs_ represent the observed pattern, while Y_Calc_ denotes the calculated pattern. An additional significant parameter is R_wp_ (Weighted Profile R-factor), which considers the statistical uncertainty in the observed data. The formula is given by Eq. (S3):

*R_wp_* =100x( $\sqrt{\frac{\sum w_{i}.{(Y_{Obs}- Y_{Calc})}^{2}}{\sum w_{i}.Y_{Obs}^{2}}}$) (S3),

here, w_i_ denotes the weight assigned to each data point, which may also be articulated about the standard deviation of the observed intensity (σ_i_). Thus, w_i_ can be expressed as (1/σ^2^). The third R-factor is the Rₑₓₚ (Expected R-factor), which denotes the optimal fit attainable considering the statistical errors contained in the data. Refer to Eq. (S4);

*R_exp_* =100x( $\sqrt{\frac{N-P}{\sum w_{i}.Y_{Obs}^{2}}}$) (S4).

The final and most critical criterion is χ² (Goodness-of-Fit, GOF). This factor provides the ultimate measure of the model's fit to the data about experimental errors; the value of χ² should not be less than one, as the one value states a 100% Perfect fit between the theoretical curve and the experimental curve, and is deemed acceptable between 1 and 3. This factor is expressed in terms of R_wp_ and R_wp_ as shown in Eq. (S5);

χ² = ${(\frac{R_{wp}}{R_{exp}})}^{2}$ (S5).

The thermal factor is a very important quantity. Inside the unit cell, the atoms vibrate around their equilibrium positions. The thermal factor represents the amplitude of the vibration. Here, we have two types of thermal factors, isotropic and anisotropic thermal factors. The isotropic one indicates that the vibration of the atom around its equilibrium position is homogeneous, as the amplitude in all directions is the same, meaning that the motion will be spherical. In this case, the thermal factor equation is:

B = 8π <u^2^> (S6).

Where B is the atomic scattering factor and <u²> is the mean square displacement of the atom (in Å²). In the anisotropic one, the vibration around the equilibrium position for the atom is not equal in the whole directions. In this case, it is described by a symmetric 3x3 tensor (B_ij_) with six independent components (B_11_, B_22_, B_33_, B_12_, B_13,_ and B_23_). So, the Temperature factor will be modified to be:

B_ij_ (hkl) = $e^{-(B_{11}h^{2}+B_{22}k^{2}+B_{33}l^{2}+2B_{12}hk+2B_{13}hl+2B_{23}kl)}$ (S7).

The previous equation illustrates the thermal factor as a function of the peak direction (hkl), which means that every peak in the XRD pattern has its thermal vibration around its equilibrium position.

As for the electron density ρ(x,y,z) at a place (x,y,z) within the unit cell can be computed mathematically (See Eq. S8)

*ρ(x,y,z) =* $\frac{1}{V}$ $\sum_{H} F\left( H_{hkl} \right).e^{-2\pi i(H.(x,y,z))}$ (S8),

*V* represents the volume of the unit cell, while *F(H_hkl_)* is the structural factor corresponding to the Miller indices.
